# Effects of auditory sensory stimulation on consciousness and physiological parameters among individuals with disorders of consciousness: a systematic review and meta-analysis

**DOI:** 10.3389/fnins.2026.1879145

**Published:** 2026-10-28

**Authors:** Jinqin Zhang, Xinyue Yan, Fubiao Huang, Yudi Huang, Jiawen Wang, Ying Zhang, Takamichi Taniguchi

**Affiliations:** 1 Department of Occupational Therapy, China Rehabilitation Research Center, Beijing, China; 2 Graduate School of Health and Welfare Sciences, International University of Health and Welfare, Otawara City, Japan; 3 Faculty of Rehabilitation, Capital Medical University, Beijing, China; 4 Rehabilitation Therapeutics, School of Nursing, Jilin University, Changchun, China; 5 Faculty of Rehabilitation Medicine, Wenzhou Medical University, Wenzhou, China; 6 Faculty of Rehabilitation, Shandong University of Traditional Chinese Medicine, Jinan, China; 7 School of Acupuncture and Moxibustion and Massage, Shandong University of Traditional Chinese Medicine, Jinan, China; 8 School of Rehabilitation Medicine, Shandong Second Medical University, Weifang City, Shandong Province, China; 9 Department of Occupational Therapy, School of Health Sciences, International University of Health and Welfare, Otawara City, Japan

**Keywords:** auditory sensory stimulation, disorders of consciousness, meta-analysis, physiological parameters, systematic review

## Abstract

**Objective:**

To systematically evaluate the effects of auditory sensory stimulation (SS) on consciousness levels and physiological parameters in patients with disorders of consciousness (DOC). Furthermore, this study aimed to explore intervention characteristics based on the frequency, intensity, time, and type (FITT) framework that may influence treatment effects and to investigate potential sources of between-study heterogeneity.

**Methods:**

This systematic review and meta-analysis was conducted in accordance with the Preferred Reporting Items for Systematic Reviews and Meta-Analyses (PRISMA) guidelines. The PubMed, Embase, Cochrane Library, Web of Science, Scopus, and ProQuest databases were systematically searched from database inception to April 25, 2026. Fifteen randomized controlled trials involving 713 patients with DOC were included. Meta-analyses were performed using postintervention endpoint data (means, standard deviations, and sample sizes), and effect sizes are expressed as the mean differences (MDs) with 95% confidence intervals (CIs). Subgroup analyses based on the FITT framework, univariable random-effects meta-regression analyses, and GRADE assessments were conducted to explore potential sources of heterogeneity and evaluate the certainty of evidence.

**Results:**

Auditory SS was associated with higher postintervention Glasgow Coma Scale (GCS) scores and a lower postintervention pulse rate than control conditions. No statistically significant between-group differences were detected in systolic blood pressure, diastolic blood pressure, respiratory rate, oxygen saturation, or body temperature. Exploratory FITT-based subgroup analyses showed numerical differences in effect estimates across some intervention categories, but these findings did not establish an optimal intervention protocol. Univariable meta-regression analyses identified no statistically significant study-level moderators. According to the GRADE assessment, the certainty of evidence was low for GCS scores and very low for all physiological outcomes.

**Conclusion:**

Auditory SS may have potential as an adjunctive rehabilitation intervention for patients with DOC, although confidence in its effect on GCS scores is limited by the low certainty of evidence. Its effects on physiological outcomes remain highly uncertain. The available evidence is insufficient to establish an optimal FITT-based protocol. Future high-quality randomized controlled trials using standardized auditory SS protocols, comprehensive reporting of stimulation parameters (particularly stimulation intensity), and consistent outcome measures are needed to establish the optimal FITT prescription for auditory SS.

## 1 Introduction

Disorders of consciousness (DOC) encompass pathological alterations in states of consciousness resulting from structural or functional damage to the neural networks governing arousal and awareness. These disorders include coma, the vegetative state (VS; also known as unresponsive wakefulness syndrome (UWS)), and the minimally conscious state (MCS), all of which are defined by standardized diagnostic criteria (Jennett and Plum, 1972; Multi-Society Task Force on PVS, 1994; Giacino et al., 2002; Laureys et al., 2010; Bruno et al., 2011).

Previous research has indicated that the incidence of VS/UWS ranges from 525 cases per million population (PMP) in various countries. Previous research has also suggested that the prevalence of MCSs is tenfold greater than that of VS (Ní Lochlainn et al., 2013). DOC significantly affects patients' survival rates, ability to perform activities of daily living (ADL), and quality of life, resulting in considerable caregiving demands and substantial societal medical resource expenditures. Individuals with DOC may experience changes in their physiological responses based on the disease, surgical procedures, therapeutic interventions, and environmental factors. These changes can include increased body temperature, cardiac output, cardiac contractility, pulse rate, and respiratory rate. These alterations can also further increase body temperature, increase cardiac output, and increase cardiovascular workload (Bailey, 2010; Yousefi et al., 2015). Improper management of these physiological changes could lead to reinjury of cerebral blood vessels and exacerbate brain damage, thereby possibly worsening the patient's level of consciousness (Vahedian-Azimi et al., 2014; Yeganeh et al., 2017). Therefore, maintaining haemodynamic stability in patients is critically important.

The brainstem, which encompasses the medulla oblongata, pons, and midbrain, is a critical structure for sustaining basic states of consciousness and plays a particularly vital role in regulating arousal and consciousness levels. This structure collaborates with the thalamus, cortex, and ascending reticular activating system (ARAS) to ensure wakefulness and the perception of external stimuli. Moreover, the brainstem is an integral part of the hypothalamic–brainstem–autonomic (HBA) axis, a crucial neural and neuroendocrine pathway that integrates signals from the hypothalamus, brainstem, and peripheral autonomic (sympathetic and parasympathetic) nerves to manage autonomic activity, metabolism, and stress responses (Sihvonen et al., 2017). The HBA axis is central to maintaining homeostasis, overseeing cardiovascular regulation (including blood pressure and heart rate), respiratory control, thermoregulation, and stress responses. Therefore, there is a complex relationship between levels of consciousness and physiological indicators in patients with consciousness disorders, necessitating concurrent attention.

The primary goal of neurological rehabilitation for patients with DOC is to increase their level of consciousness. It is also essential to control fluctuations in physiological parameters that could further compromise cerebrovascular integrity. While pharmacological agents and neuromodulatory interventions remain the cornerstone treatments, noninvasive approaches such as sensory stimulation have emerged as complementary therapeutic modalities. A longstanding theoretical perspective suggests that regulated sensory stimulation may facilitate positive therapeutic outcomes; however, patients exposed to undifferentiated bombardment of sensory information lose information processing ability owing to habituation (Wood et al., 1992). Rehabilitation therapists, who are proficient in sensory modulation principles, can provide controlled sensory stimuli calibrated to higher thresholds of reticular neurons, thereby increasing cortical activity (Lannin et al., 2013).

Auditory sensory stimulation is more critical than other sensory stimuli (e.g., visual, verbal, motor functions, etc.) for the following reasons: on the one hand, auditory function is the last perceptual function that coma patients lose (Grimm and Kreutz, 2018); on the other hand, auditory sensory stimulation is simple to implement, requires no active cooperation from patients, and is applicable to various types of patients with DOC. Although auditory SS has been widely used in clinical occupational therapy practice, there is significant heterogeneity in specific implementation protocols. Existing randomized controlled studies have explored the rehabilitation efficacy of different types of auditory sensory stimulation for patients with DOC; however, clinical evidence regarding their therapeutic effects remains inconsistent (Goudarzi-Rad et al., 2023; Xiao et al., 2023). Although previous studies have conducted meta-analyses on the efficacy of sensory stimulation and family-centered sensory stimulation therapy for patients with DOC, these analyses suffer from limitations such as insufficient timeliness, lack of refined classification of sensory stimulation types, and failure to examine the impact of different intervention types and dosages on the rehabilitation outcomes of auditory SS (Padilla and Domina, 2016; Zuo et al., 2021). To our knowledge, no systematic review has comprehensively investigated the effects of auditory SS on the consciousness levels and physiological indicators of patients with DOC.

## 2 Aims and objectives

We aimed to address the following research questions:

(1) What are the effects of auditory sensory stimulation on consciousness levels and physiological parameters in patients with disorders of consciousness?
(2) Are differences in the characteristics of an auditory stimulation protocol characteristics associated with differences in its effect on consciousness levels?

This is the first meta-analysis to evaluate the impact of auditory sensory stimulation on the consciousness levels and physiological indicators of patients with DOC, thereby providing new perspectives for nonpharmacological interventions aimed at promoting arousal in this patient population.

## 3 Methodology

### 3.1 Registration

The systematic review and meta-analysis were performed in accordance with the Preferred Reporting Items for Systematic Reviews and Meta-Analyses (PRISMA) guidelines and the Cochrane Handbook recommendations (Higgins and Cochrane Collaboration, 2019). It was also performed in accordance with the Population, Interventions, Comparisons, Outcomes, and Study Design (PICOS) framework (Page et al., 2021). This review was registered a priori with the International Prospective Register of Systematic Reviews (PROSPERO) (CRD420251022357; Efficacy of Auditory Sensory Stimulation on Consciousness Levels and Physiological Parameters in Patients with Disorder of Consciousness: A Systematic Review and Meta-Analysis). During registration, we submitted the protocol on the website. The protocol submitted during registration can be found in the Supplementary Material 1. In the actual meta-analysis, we additionally incorporated PEDro analysis.

### 3.2 Information sources and search strategy

We searched the PubMed, Embase, Cochrane, Web of Science, Scopus, and ProQuest databases from inception to April 25, 2026, to identify English-language studies. The search strategy employed a combination of controlled vocabulary terms (MeSH terms for PubMed and Emtree terms for Embase) with free-text terms; the primary search terms included consciousness disorder, acoustic stimulation, randomized controlled trials, etc. The detailed search strategy is provided in Supplementary Material 2. Additionally, we manually screened the reference lists of relevant articles and review papers and searched the Google Scholar database to identify additional eligible publications.

### 3.3 Inclusion and exclusion criteria

#### 3.3.1 Inclusion criteria

(1) Study type: Only randomized controlled trials (RCTs) were eligible. Baseline characteristics must have demonstrated comparability between the intervention and control groups. Studies were restricted to English-language publications.
(2) Participants: Patients with any etiology of impaired consciousness.
(3) Interventions: The experimental group received auditory SS (any protocol) in addition to standard care, whereas the control group received either no auditory intervention or routine environmental sound exposure.
(4) Outcomes: Primary outcome: Glasgow Coma Scale (GCS) scores. The secondary outcomes included physiological parameters such as temperature (Temp), systolic/diastolic blood pressure (SBP/DBP), oxygen saturation (SPO_2_), pulse rate (PR), and respiratory rate (RR).

#### 3.3.2 Exclusion criteria

(1) Non-RCTs (e.g., surveys, descriptive studies, meta-analyses, systematic reviews, preclinical research, or study protocols).
(2) Insufficient data were provided for quantitative analysis.
(3) Duplicate publications.
(4) Unavailable as full-text articles.
(5) Have fewer than 10 participants (small sample size threshold).

### 3.4 Study screening

After the retrieved studies were imported into the reference management software NoteExpress, a unified screening process was conducted. Two researchers independently and preliminarily screened the studies on the basis of the inclusion and exclusion criteria. The titles and abstracts were subsequently screened to exclude articles that were clearly irrelevant. The full texts of the remaining studies were downloaded and screened to determine their eligibility for inclusion. The two researchers performed cross-checking. Disagreements regarding study inclusion were resolved via discussion and by consulting a third party.

### 3.5 Data extraction

Two researchers independently extracted the data into a predesigned Excel spreadsheet. The following data were extracted: general information (first author, publication year, country, study type, multicentre status, sample size), patient characteristics (gender, age, disease type, baseline features), intervention methods (control group and experimental group interventions with specific details), outcome measures (GCS scores, SBP, DBP, RR, SPO_2_, PR, temperature), and relevant study findings. We extracted all relevant outcome indicator data from the two measurement time points at baseline and post-intervention for the included RCTs. For the relevant outcomes, we extracted data from both the baseline and postintervention phases. The baseline data were used to assess the comparability between groups, while the postintervention means and standard deviations were used for quantitative analyses. Any discrepancies arising during the data extraction process were resolved through discussion or by consulting a third party for final determination.

### 3.6 Quality assessment and risk of bias assessment

Two researchers independently evaluated the methodological rigor of the included RCTs by using the PEDro scale (Paci et al., 2022). This scale consists of 11 items that evaluate baseline comparability, randomization, blinding, allocation concealment, follow-up, intention-to-treat analysis, and statistical reporting. Each item was scored as either ‘yes’ or ‘no’. Items scored as ‘yes’ were assigned a score of one point, whereas those marked ‘no’ were assigned a score of zero points. The established guidelines of the PEDro scale were applied to determine the cumulative score for each article. The first item was excluded from the score because of its relevance to the RCT's external validity. Consequently, the methodological quality of each RCT was evaluated using items 2–11, which pertain to the internal validity of the RCT. The scores of items 2–11 are summed to obtain the total PEDro score. The RCTs were classified according to their methodological quality, with a PEDro score of 0–3 points considered low, 4–5 points considered average, 6–8 points considered good, and 9–10 points considered very good. Disagreements between the results of the two researchers were resolved through discussion or by consulting a third researcher.

Two researchers independently assessed the risk of bias for each RCT by using the Cochrane ROB 2.0 tool. The RoB 2 assesses 5 domains of bias: (1) bias arising from the randomization process; (2) bias due to deviations from intended interventions; (3) bias due to missing outcome data; (4) bias in measurement of the outcome; and (5) bias in selection of the reported result. Disagreements between the results of the two researchers were resolved through discussion or by consulting a third researcher.

### 3.7 Data analysis and synthesis

Descriptive statistics were used to summarize the study characteristics. Continuous data are reported as the mean (standard deviation [SD]) or median (first‒third quartile [Q]), as appropriate. Nominal and categorical variables are summarized as counts and percentages.

#### 3.7.1 Effect measures

Data analysis was conducted using Review Manager 5.4 in accordance with the relevant statistical methods. For continuous outcomes, postintervention mean values, standard deviations (SDs), and sample sizes from the intervention and control groups were extracted and entered into the meta-analysis. In studies that reported standard errors (SEs) of group means rather than SDs, SDs were calculated using the formula SD = SE × √*n*, where *n* represents the number of participants analysed in the corresponding group at the selected assessment time point. Since the included studies within each meta-analysis used the same measurement scales, effect sizes were expressed as the mean differences (MDs) with 95% confidence intervals (95% CIs).

Baseline data were extracted to assess the comparability between the intervention and control groups before treatment but were not used for calculating change scores. Although change-from-baseline analysis is recommended in meta-analyses, the included studies did not consistently report change-score standard deviations or correlation coefficients between baseline and postintervention measurements. Therefore, endpoint values after intervention were used for quantitative synthesis to ensure consistency across studies and avoid assumptions regarding missing statistical parameters.

#### 3.7.2 Assessment of heterogeneity

*I*^2^ values were calculated to quantify the degree of heterogeneity. An *I*^2^ < 25% indicates a low degree of heterogeneity between the study results; an I^2^ between 25% and 50% indicates a moderate degree of heterogeneity between the study results; and an *I*^2^ > 50% indicates a high degree of heterogeneity between the study results (Higgins et al., 2003).

#### 3.7.3 Meta-analyses

The meta-analysis was conducted using Review Manager 5.4. An *I*^2^ value of 50% was used as an operational threshold for model selection. When the *I*^2^ value was ≤ 50% and no substantial clinical or methodological heterogeneity was identified, the fixed-effects model was applied to pool the effect sizes. When the *I*^2^ value was > 50% or when important clinical or methodological heterogeneity was considered to exist, the random-effects model was used for pooled analysis (Borenstein et al., 2010).

#### 3.7.4 Subgroup analyses

Subgroup analyses were performed to assess sources of heterogeneity with respect to the primary outcome, i.e., the GCS score. These analyses were based on the intervention type, initiation time of the intervention, intervention frequency, duration of intervention, disease type, types of control group interventions, and intervention duration per session. The threshold for statistical significance was a *p-*value less than 0.05.

#### 3.7.5 Sensitivity analyses

Furthermore, sensitivity analyses were conducted via the leave-one-out method to determine whether the results were influenced by any single trial. When the MD, *p*-value, 95% CI, or heterogeneity showed substantial changes after excluding a particular study, the study was considered to have an effect on the robustness of the results.

#### 3.7.6 Meta-regression analysis

To explore the potential sources of heterogeneity in the primary outcome (GCS score), univariable random-effects meta-regression analyses were performed using Stata 17.0 based on prespecified moderator variables. The candidate moderators included risk of bias, intervention type, intervention frequency, intervention duration, intervention duration per session, disease type, and types of control group interventions. Each moderator was entered into a separate regression model independently. Categorical moderators with more than two categories were coded using dummy variables, with a reference category specified a priori. Between-study variance was estimated using the restricted maximum likelihood (REML) method, and standard errors were adjusted using the Knapp–Hartung method. The results are reported as regression coefficients (*β*), 95% CIs, *p*-values, residual *I*^2^ values, and adjusted *R*^2^ values. Given the limited number of included studies, all meta-regression analyses were considered exploratory analyses. Owing to the limited statistical power and the risk of overfitting, multivariable meta-regression analyses were not performed.

#### 3.7.7 Publication bias

If more than 10 studies reported an outcome, the risk of publication bias was analysed through a funnel plot. The effect size serves as the horizontal coordinate, and the standard error serves as the vertical coordinate to plot a scatter diagram, visually assessing the symmetry of the graph. In the absence of publication bias, studies should exhibit an inverted funnel-shaped symmetrical distribution. If significant asymmetry is observed (e.g., small-sample studies clustering on one side of the graph), it may indicate potential publication bias.

#### 3.7.8 Certainty of evidence

The certainty of the evidence was assessed independently by two reviewers using the Grading of Recommendations, Assessment, Development and Evaluation (GRADE) framework (Guyatt et al., 2008). Since only RCTs were included in this review, five downgrading factors were considered: risk of bias, inconsistency, indirectness, imprecision and publication bias. The strength of the evidence was rated as “high,” “moderate,” “low,” or “very low.” Any differences were resolved by consulting a third researcher.

## 4 Results

### 4.1 Search results

A total of 4,103 records were identified through a systematic search of six databases. After 1,063 duplicate records were removed, 3,040 records remained for title and abstract screening. Of these, 2,998 records were excluded because they did not meet the eligibility criteria. The full texts of the remaining 42 reports were retrieved and assessed for eligibility. Following full-text assessment, 27 reports, including review articles or meta-analyses (*n* = 3), case reports (*n* = 2), study protocols (*n* = 1), non-English-language reports (*n* = 3), non-randomized studies (*n* = 3), studies with insufficient data for quantitative synthesis (*n* = 5), and studies that did not address the review question (*n* = 10), were excluded. Ultimately, 15 studies were included in the systematic review and meta-analysis. The detailed study-selection process is presented in Figure 1.

**Figure 1:**
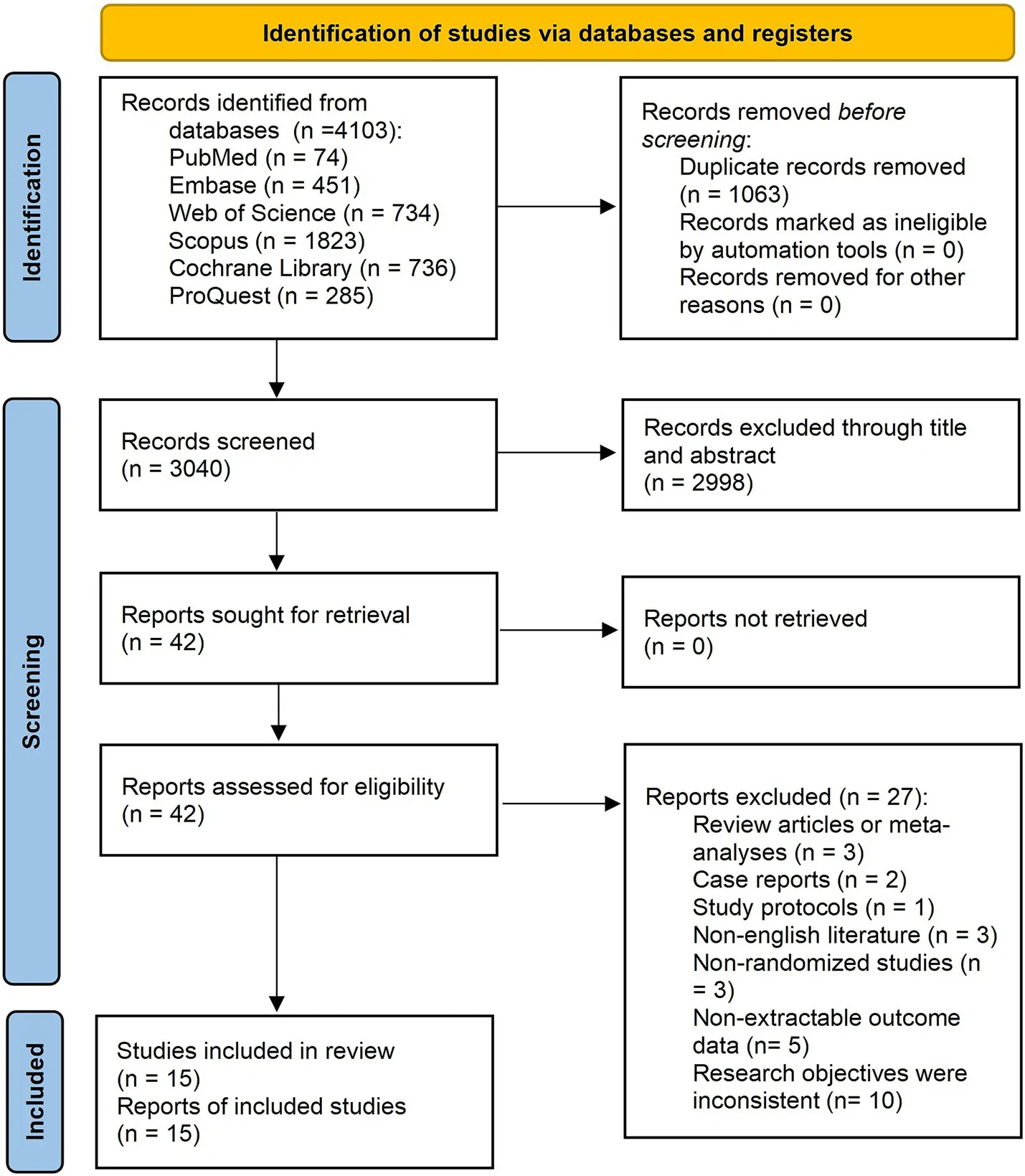
PRISMA flow chart.

### 4.2 Characteristics of the included studies

Ultimately, 15 articles (Giesbrecht Puggina et al., 2011; Parveen et al., 2015; Tavangar et al., 2015; Moghaddam et al., 2016; Hoseinzadeh et al., 2017; Çevik and Namik, 2018; Bahonar et al., 2019a, 2019b; Mohammadi et al., 2019; Froutan et al., 2020; Varghese et al., 2021; Yekefallah et al., 2021; Hoseini et al., 2022; Goudarzi-Rad et al., 2023; Xiao et al., 2023) involving 713 patients were included in the meta-analysis. A total of 358 patients with DOC were assigned to the experimental group and received auditory SS. The remaining 355 patients with DOC composed the control group and received regular stimulation or no auditory intervention (Supplementary Table 1).

### 4.3 Quality assessment and risk of bias assessment

Eight RCTs scored 7/10 on the PEDro scale, and six RCTs scored 8/10, indicating that the quality of these RCTs was good. One RCT scored 9/10, indicating that its quality was very good (Supplementary Table 1).

After assessing the risk of bias of the included studies, we identified 8 studies with a low risk of bias, whereas 7 studies were found to have a high risk of bias. Figure 2 displays the proportion chart of quality assessment items in this study. Figure 3 shows a schematic diagram of the methodological quality evaluation for the studies included in this research.

**Figure 2:**
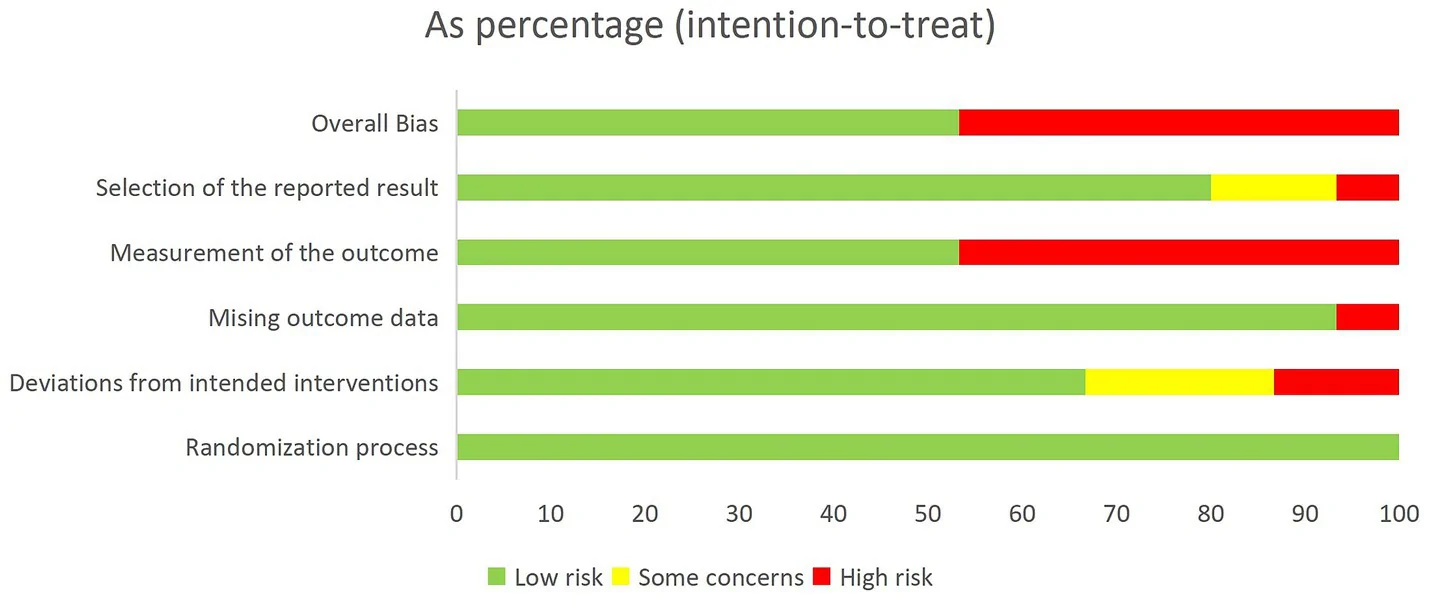
Risk of bias graph.

**Figure 3:**
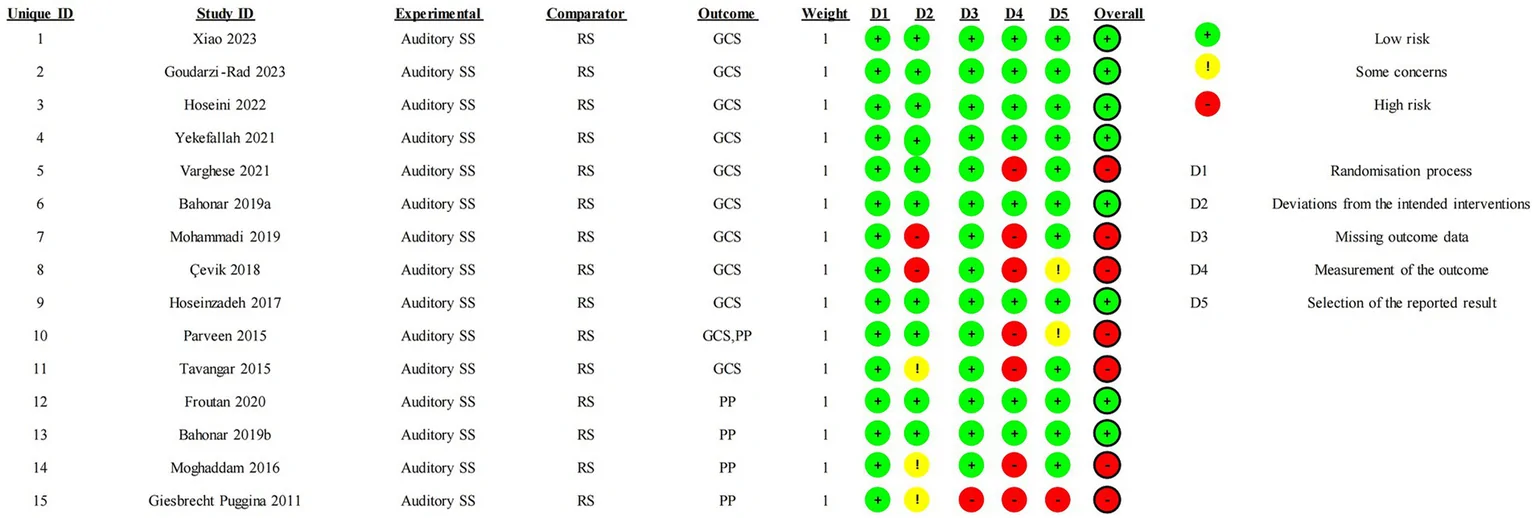
Risk of bias summary. Auditory SS = auditory sensory stimulation. RS=routine stimulation. GSC=Glasgow Coma Scale, PP=physiological parameters.

### 4.4 Meta-analysis results

#### 4.4.1 Level of consciousness for the GCS

Eleven RCTs (Tavangar et al., 2015, Parveen et al., 2015; Hoseinzadeh et al., 2017; Çevik and Namik, 2018; Bahonar et al., 2019a; Mohammadi et al., 2019; Varghese et al., 2021; Yekefallah et al., 2021; Hoseini et al., 2022; Goudarzi-Rad et al., 2023; Xiao et al., 2023) were conducted to evaluate the effects of various auditory SS interventions on the GCS scores of patients with DOC. There was a significant amount of heterogeneity among the studies (*I*^2^ = 84% > 50%, and *p* < 0.1 for the *Q* test); therefore, the random effects model was employed for pooled analysis. The pooled MD was 2.29 (95% CI: 1.75–2.82). The *Z* score was 8.35 (*p* < 0.05), indicating a statistically significant difference between the experimental and control groups. These results demonstrated that auditory stimuli could significantly improve the GCS scores of patients with DOC (Figure 4). The certainty of evidence regarding the increase in consciousness levels in patients with DOC through various auditory SS interventions was low (Supplementary Table 2).

**Figure 4:**
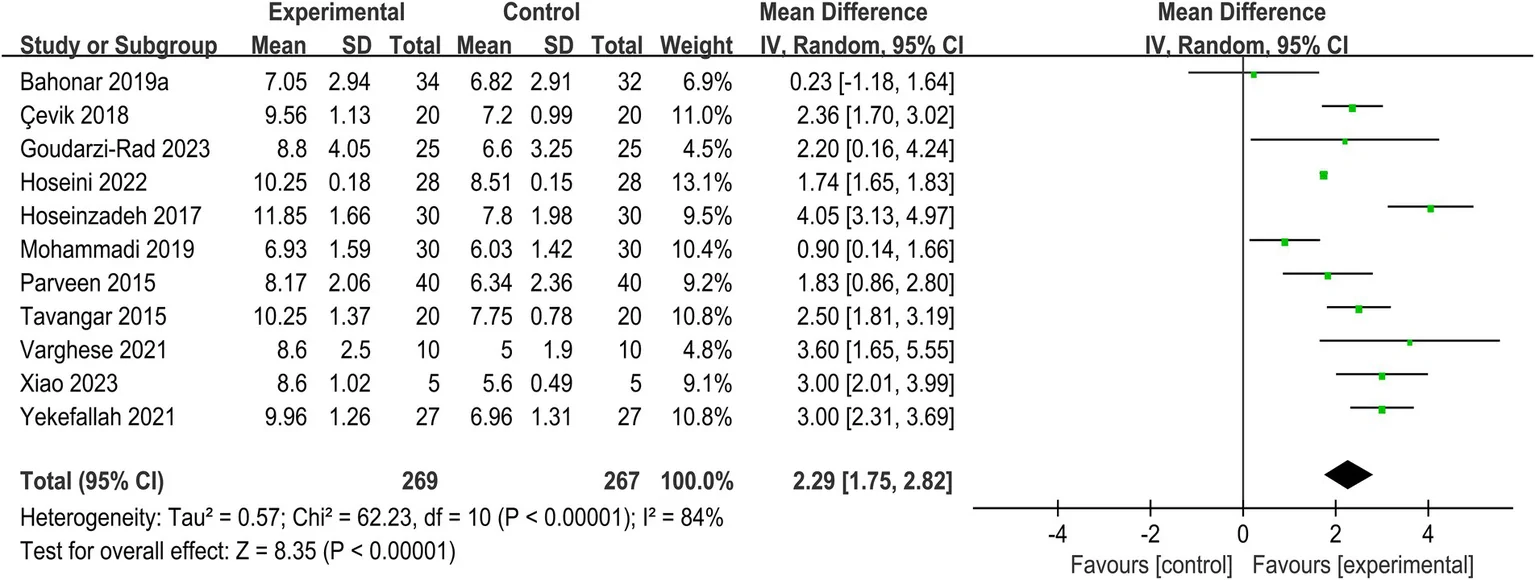
Summarizing the results of GCS.

#### 4.4.2 SBP

Five RCTs (Giesbrecht Puggina et al., 2011; Parveen et al., 2015; Moghaddam et al., 2016; Bahonar et al., 2019b; Froutan et al., 2020) reported the effects of auditory sensory stimulation on SBP in patients with DOC. Heterogeneity analysis revealed an *I*^2^ statistic of 72%, exceeding the 50% threshold, therefore, there was a high degree of heterogeneity among the selected studies. Consequently, a random effects model was employed for the meta-analysis. The pooled MD across the five studies was −4.47 (95% CI: −10.51 to 1.57), with a z score of 1.45 and a *p*-value of 0.15, suggesting no statistically significant difference. The results demonstrated that auditory sensory stimulation does not significantly improve SBP levels in patients with DOC (Figure 5). The certainty of evidence for the estimated effect of auditory SS on SBP in patients with DOC was very low (Supplementary Table 2).

**Figure 5:**
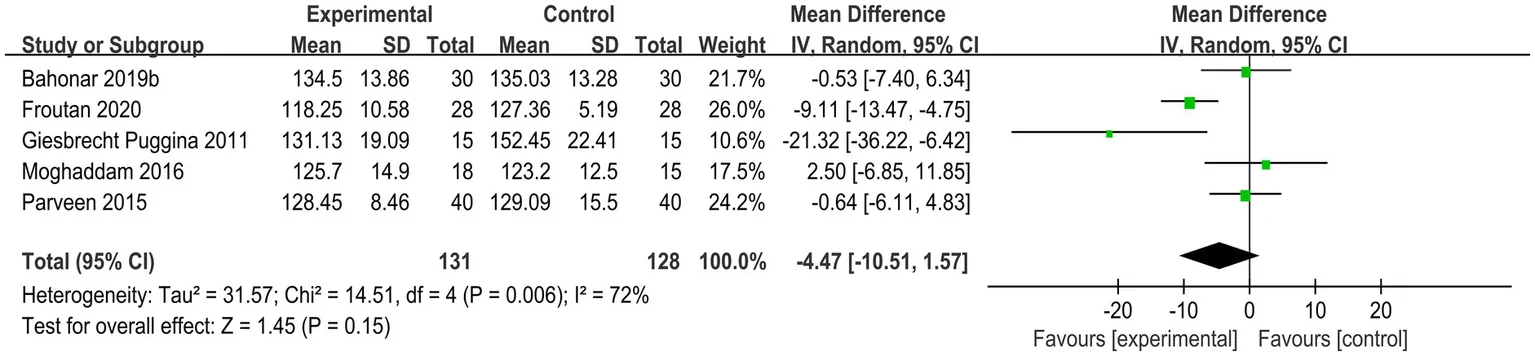
Summarizing the results of SBP.

#### 4.4.3 DBP

Five RCTs (Giesbrecht Puggina et al., 2011; Parveen et al., 2015; Moghaddam et al., 2016; Bahonar et al., 2019b; Froutan et al., 2020) reported the effects of auditory sensory stimulation on DBP in patients with DOC. Heterogeneity analysis revealed a moderate degree of heterogeneity (*I*^2^ = 49%). The results of the remaining five studies were pooled using a fixed effects model. The meta-analysis yielded an MD of −0.65 (95% CI: −2.78 ~ 1.49; *p* = 0.55; *I*^2^ = 49%), indicating no statistically significant difference. After one study by Froutan et al. was excluded via sensitivity analysis, the degree of heterogeneity significantly decreased (*I*^2^ = 0%). These findings suggest that auditory sensory stimulation does not significantly improve DBP levels in patients with DOC (Figure 6). The certainty of evidence for the estimated effect of auditory SS on DBP in patients with DOC was very low (Supplementary Table 2).

**Figure 6:**
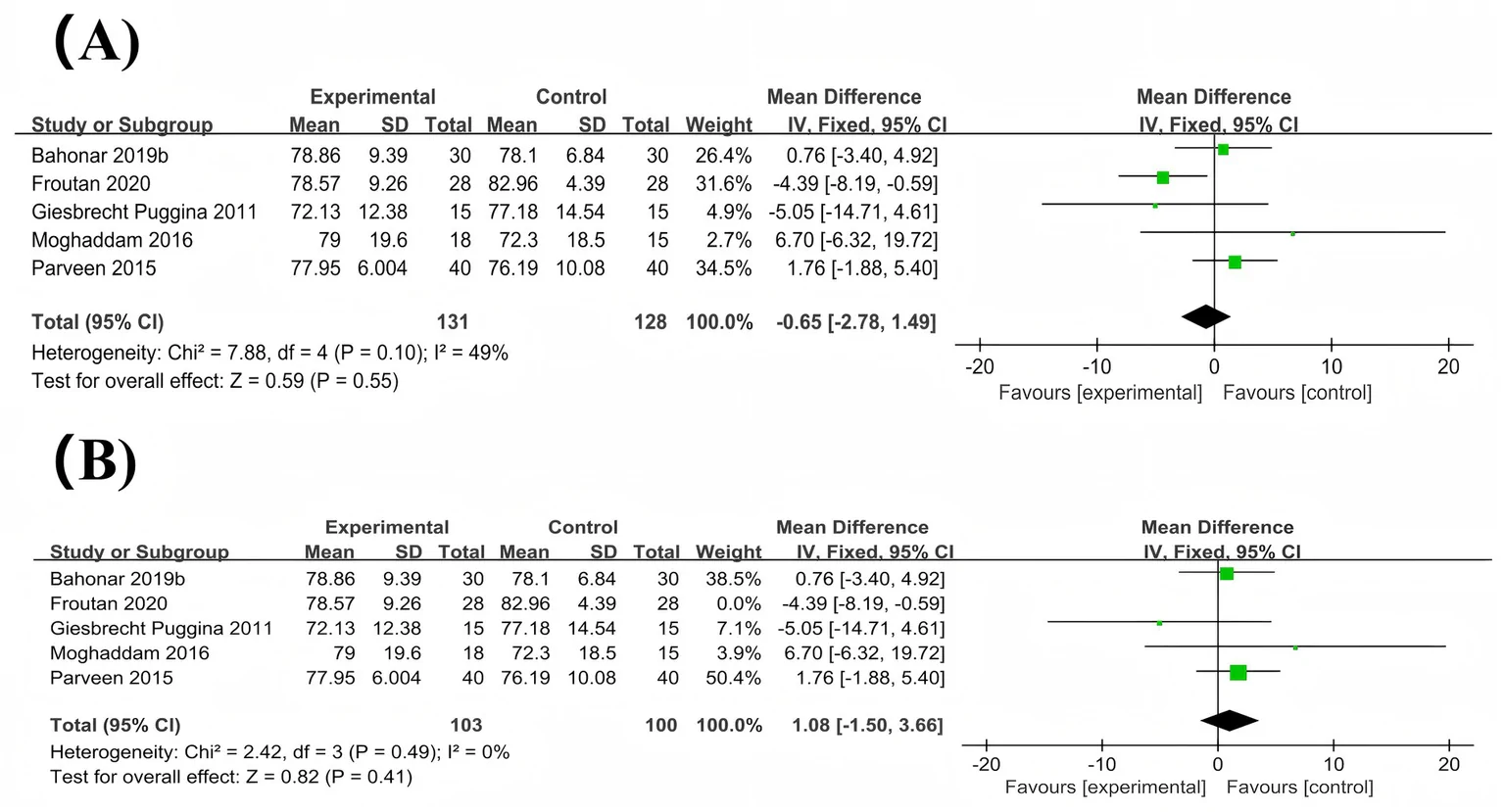
Summarizing the results of DBP. **(A)** Primary analysis including all eligible studies. **(B)** Sensitivity analysis excluding Froutan et al. (2020).

#### 4.4.4 RR

Four RCTs (Giesbrecht Puggina et al., 2011; Parveen et al., 2015; Moghaddam et al., 2016; Froutan et al., 2020) reported the effect of auditory sensory stimulation on RR in patients with DOC. The heterogeneity analysis yielded an *I*^2^ of 89%, indicating a high degree of heterogeneity. The results of the four studies were pooled using a fixed effects model. The meta-analysis yielded an MD of −0.86 (95% CI: −5.02 to 3.29; *p* = 0.68; *I*^2^ = 89%), indicating that there was no statistically significant difference. After sensitivity analysis was performed and one study by Froutan et al. was excluded, the degree of heterogeneity was significantly lower (*I*^2^ = 0%). These findings suggest that auditory sensory stimulation does not significantly improve RR levels in patients with DOC (Figure 7). The certainty of evidence for the estimated effect of auditory SS on RR in patients with DOC was very low (Supplementary Table 2).

**Figure 7:**
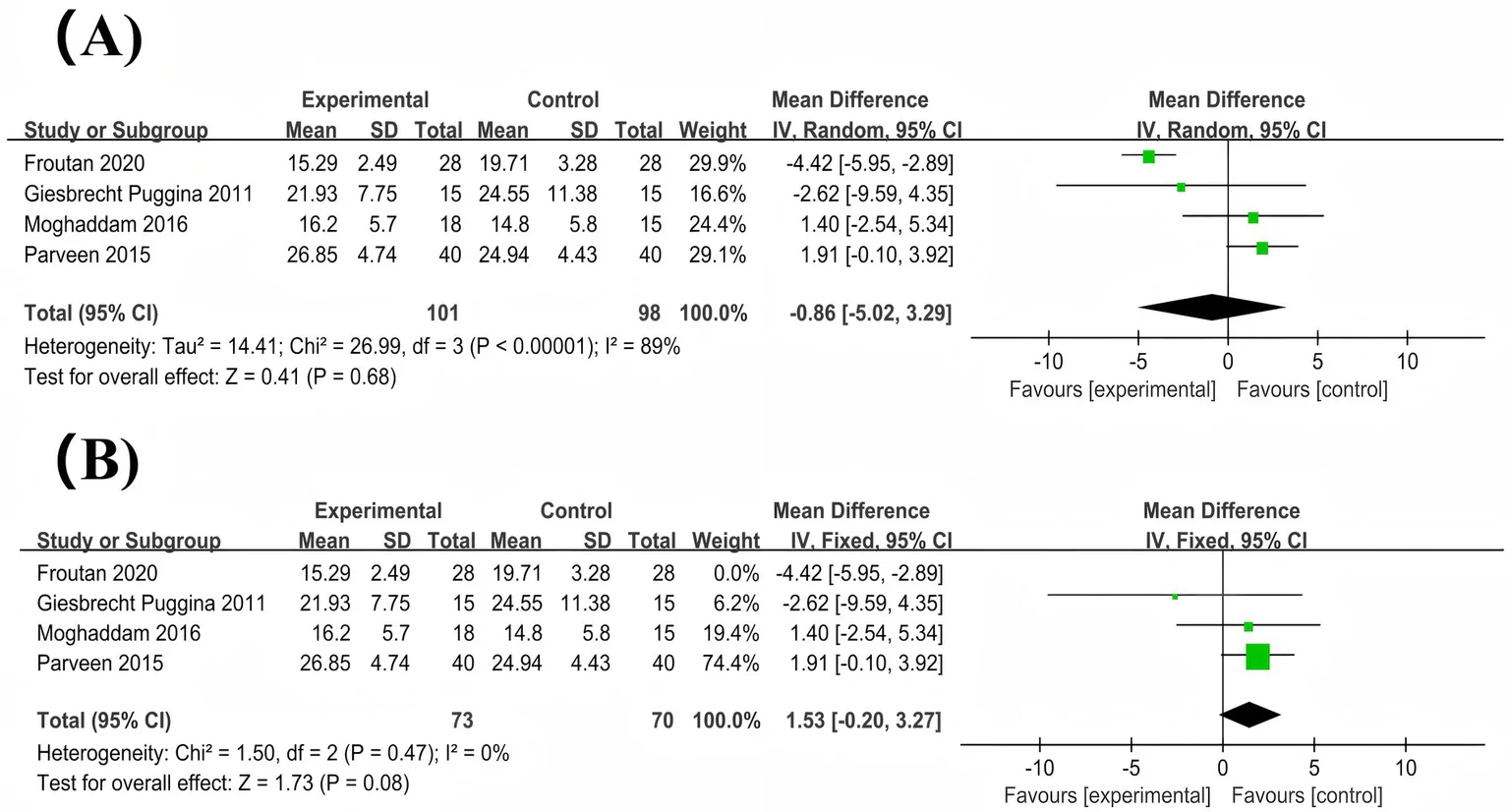
Summarizing the results of RR. **(A)** Primary analysis including all eligible studies. **(B)** Sensitivity analysis excluding Froutan et al. (2020).

#### 4.4.5 SPO_2_

Four RCTs (Giesbrecht Puggina et al., 2011; Parveen et al., 2015; Moghaddam et al., 2016; Froutan et al., 2020) evaluated the effect of auditory sensory stimulation on SPO_2_ in patients with DOC. The heterogeneity analysis yielded an *I*^2^ of 52%. The findings of the four studies were pooled using a fixed effects model. The meta-analysis yielded an MD of 0.61 (95% CI: −0.35 to 1.58; p = 0.21; *I*^2^ = 52%), indicating there was no statistically significant difference. After one study by Froutan et al. was excluded through sensitivity analysis, the degree of heterogeneity was significantly lower (*I*^2^ = 14%). These findings suggest that auditory sensory stimulation can not improve SPO_2_ levels in patients with DOC (Figure 8). The certainty of evidence for the estimated effect of auditory SS on SPO_2_ in patients with DOC was very low (Supplementary Table 2).

**Figure 8:**
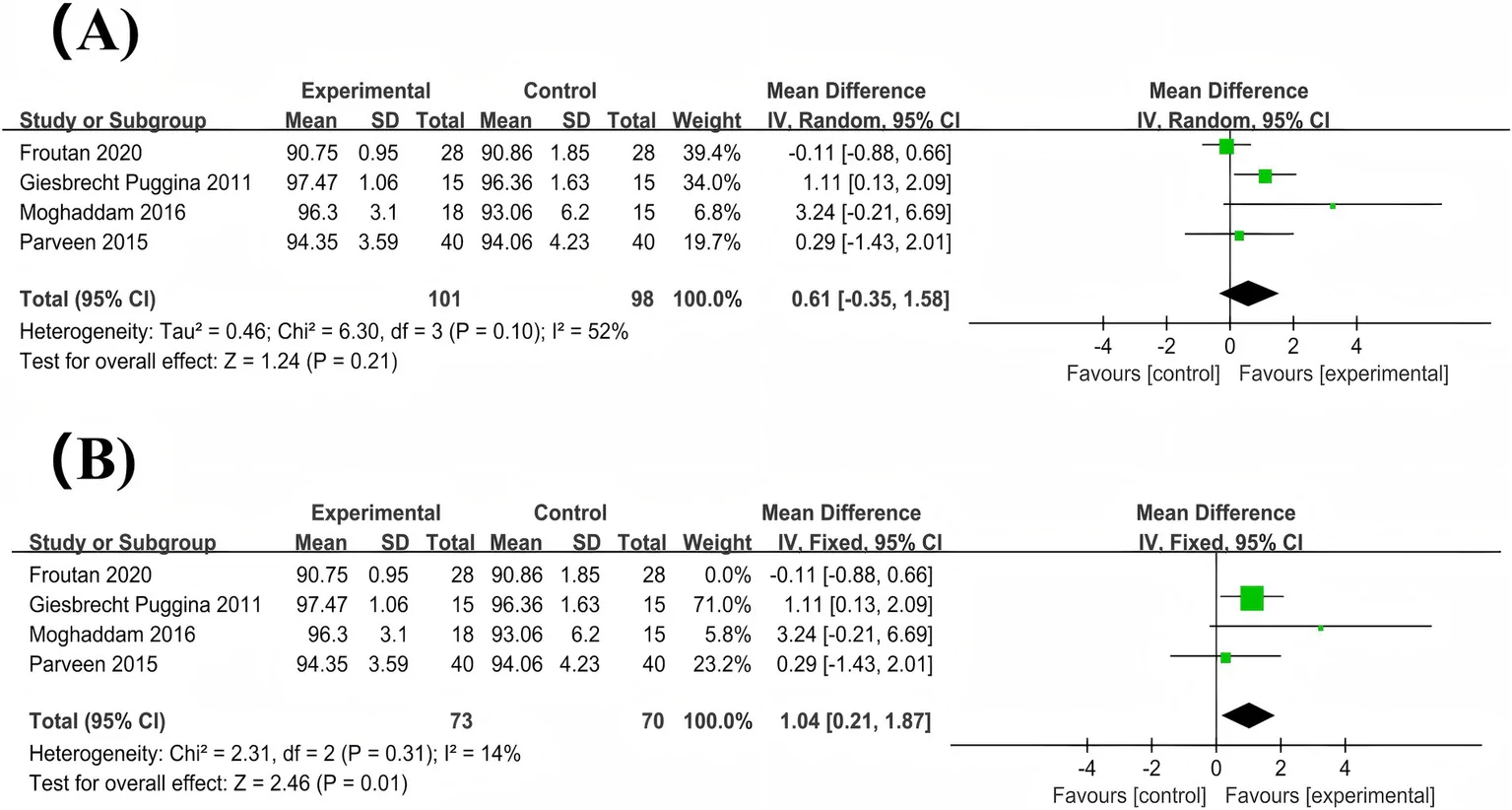
Summarizing the results of SPO_2_. **(A)** Primary analysis including all eligible studies. **(B)** Sensitivity analysis excluding Froutan et al. (2020).

#### 4.4.6 PR

Five RCTs (Giesbrecht Puggina et al., 2011; Parveen et al., 2015; Moghaddam et al., 2016; Bahonar et al., 2019b; Froutan et al., 2020) reported the effects of auditory sensory stimulation on PR in patients with DOC. The heterogeneity analysis yielded *I*^2^ = 68% > 50%. Consequently, a random effects model was employed for the meta-analysis. The pooled MD across the five studies was −9.80 (95% CI: −16.36 to −3.25), with *p* = 0.003, indicating statistically significant differences. Auditory SS was associated with a lower postintervention PR (Figure 9). After one study by Froutan et al. was excluded through sensitivity analysis, the degree of heterogeneity was significantly lower (*I*^2^ = 30%). However, the certainty of evidence regarding various auditory SS interventions being able to effectively reduce the PR of patients with DOC was very low (Supplementary Table 2).

**Figure 9:**
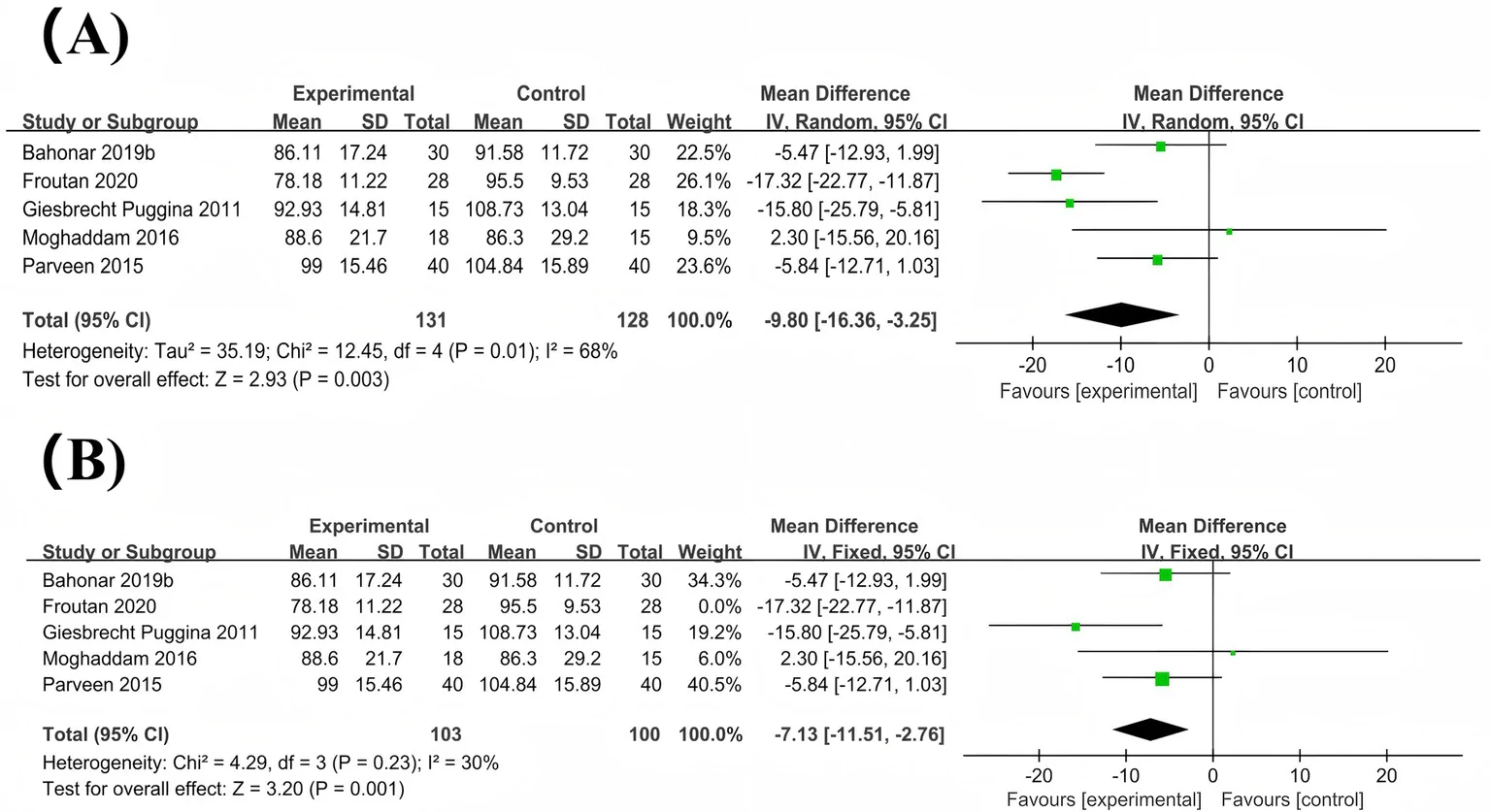
Summarizing the results of PR.

#### 4.4.7 Temperature

Three RCTs (Giesbrecht Puggina et al., 2011; Moghaddam et al., 2016; Froutan et al., 2020) reported the effects of auditory sensory stimulation on temperature among patients with DOC. The heterogeneity analysis yielded *I*^2^ = 75%>50%; therefore, there was a high degree of heterogeneity among the included studies, and a random effects model was used for the meta-analysis. The pooled MD across the three studies was −0.17 (95% CI: −0.51 to 0.18), with *p* = 0.34, indicating no statistically significant difference. The results demonstrate that auditory sensory stimulation does not significantly improve temperature among patients with DOC (Figure 10). The certainty of evidence for the estimated effect of auditory SS on Temp in patients with DOC was very low (Supplementary Table 2).

**Figure 10:**
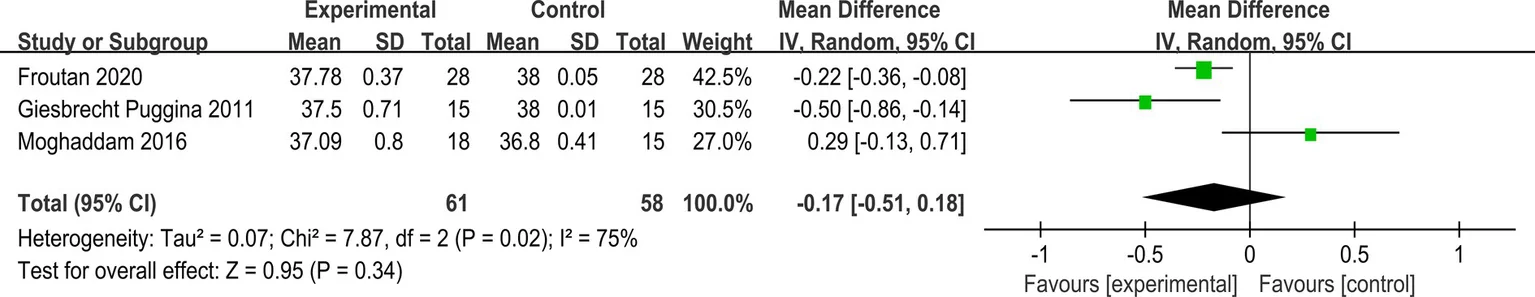
Summarizing the results of temperature.

#### 4.4.8 Subgroup analysis of the level of consciousness for the GCS

(1) Subgroup analysis was conducted on the basis of intervention type, which was divided into two subgroups: linguistic auditory stimulation and nonlinguistic auditory stimulation. Five of the included trials (Bahonar et al., 2019a; Yekefallah et al., 2021; Hoseini et al., 2022; Goudarzi-Rad et al., 2023; Xiao et al., 2023) employed nonlinguistic auditory stimulation, and six trials (Parveen et al., 2015; Tavangar et al., 2015; Hoseinzadeh et al., 2017; Çevik and Namik, 2018; Mohammadi et al., 2019; Varghese et al., 2021) utilized linguistic auditory stimulation. The pooled effect sizes were 2.11 (95% CI: 1.26 to 2.96; *p* < 0.00001) and 2.45 (95% CI: 1.58–3.32; *p* < 0.00001), respectively. The results indicated that both nonlinguistic and linguistic auditory stimulation effectively improved consciousness levels among patients with DOC, and there was no significant difference between the two subgroups (*p* = 0.59; Figure 11). According to the GRADE assessment, the certainty of evidence was very low for both nonlinguistic and linguistic auditory stimulation (Supplementary Table 2).
(2) Subgroup analysis was conducted on the basis of the initiation time of the intervention, which was divided into three subgroups: within 24 h of admission, beyond 24 h of admission, and patients for whom the intervention start time was not reported. Three of the included trials (Bahonar et al., 2019a; Mohammadi et al., 2019; Hoseini et al., 2022) initiated the intervention within 24 h of admission (MD = 1.15; 95% CI: 0.3 to 2; *p* = 0.008). Three trials (Hoseinzadeh et al., 2017; Parveen et al., 2015; Tavangar et al., 2015) implemented the intervention beyond 24 h after admission (MD = 2.79; 95% CI: 1.60–3.98; *p* < 0.00001); Five trials (Çevik and Namik, 2018; Varghese et al., 2021; Yekefallah et al., 2021; Goudarzi-Rad et al., 2023; Xiao et al., 2023) did not report the intervention start time (MD = 2.75; 95% CI: 2.34–3.16; *p* < 0.00001). These results demonstrated that auditory stimulation interventions initiated either within 24 h of admission, beyond 24 h, or at unspecified times significantly increased the level of consciousness of patients with DOC. There were highly significant differences observed among the three groups (*p* = 0.004) (Figure 12). According to the GRADE assessment, the certainty of evidence was very low for the subgroups receiving the intervention within 24 h of admission and beyond 24 h of admission, whereas the certainty of evidence was moderate for the subgroup in which the intervention start time was not reported (Supplementary Table 2).
(3) Subgroup analysis was conducted on the basis of intervention frequency, which was divided into three subgroups: intervention frequency ≤1 time/day, intervention frequency of 2 times/day, and intervention frequency of 3 times/day. Three included studies (Mohammadi et al., 2019; Yekefallah et al., 2021; Xiao et al., 2023) had an intervention frequency of ≤1 time/day, with a pooled MD of 2.29 (95% CI: 0.87–3.71; *p* = 0.002). Six studies (Parveen et al., 2015; Tavangar et al., 2015; Çevik and Namik, 2018; Bahonar et al., 2019a; Hoseini et al., 2022; Goudarzi-Rad et al., 2023) reported an intervention frequency of 2 times/day, with a pooled MD of 1.92 (95% CI: 1.46–2.39, p < 0.00001). Two studies (Hoseinzadeh et al., 2017; Varghese et al., 2021) reported an intervention frequency of 3 times/day, with a pooled MD of 3.97 (95% CI: 3.13–4.80, *p* < 0.00001). The results demonstrated that auditory stimulation intervention frequencies of ≤1 time/day, 2 times/day, or 3 times/day significantly improved levels of consciousness among patients, with highly significant differences among the three groups (*p* = 0.0001). The intervention frequency of 3 times/day resulted in more pronounced improvements in consciousness disorders (Figure 13). Intervention frequency may be one of the factors contributing to the high degree of heterogeneity in GCS scores across the 11 included studies. However, the certainty of evidence was very low for the ≤1 time/day and 2 times/day subgroups and low for the 3 times/day subgroup (Supplementary Table 2).
(4) Subgroup analysis was conducted on the basis of the duration of intervention, and the studies were divided into two subgroups: those with an intervention duration of less than 10 days and those with an intervention duration of 10 days or more. Among the included studies, four (Mohammadi et al., 2019; Varghese et al., 2021; Yekefallah et al., 2021; Hoseini et al., 2022) had an intervention duration of less than 10 days, with a pooled MD of 2.10 (95% CI: 1.23–2.96, *p* < 0.00001). Seven studies (Parveen et al., 2015; Tavangar et al., 2015; Hoseinzadeh et al., 2017; Çevik and Namik, 2018; Bahonar et al., 2019a; Goudarzi-Rad et al., 2023; Xiao et al., 2023) had an intervention duration of 10 days or more, with a pooled MD of 2.40 (95% CI: 1.67–3.13, *p* < 0.00001).The results indicated that auditory stimulation interventions, regardless of whether the duration was less than 10 days or 10 days or more, could significantly improve levels of consciousness among patients (Figure 14). There was no significant difference between the two subgroups (*p* = 0.60). According to the GRADE assessment, the certainty of evidence was very low for the subgroup with an intervention duration of less than 10 days and low for the subgroup with an intervention duration of 10 days or more (Supplementary Table 2).
(5) Subgroup analysis was conducted on the basis of disease type, and the studies were divided into two subgroups: pure traumatic brain injury and mixed disease. Seven studies (Parveen et al., 2015; Hoseinzadeh et al., 2017; Bahonar et al., 2019a; Mohammadi et al., 2019; Yekefallah et al., 2021; Hoseini et al., 2022; Goudarzi-Rad et al., 2023) examined pure traumatic brain injury, with a pooled MD of 2.03 (95% CI: 1.28–2.79; *p* < 0.00001). Four studies (Tavangar et al., 2015; Çevik and Namik, 2018; Varghese et al., 2021; Xiao et al., 2023) examined mixed diseases, with a pooled MD of 2.58 (95% CI: 2.16–3.00; *p* < 0.00001).These results demonstrated that auditory stimulation significantly improved levels of consciousness in patients with DOC caused by either pure traumatic brain injury or mixed diseases, with no significant difference between the two subgroups (*p* = 0.21) (Figure 15). However, the certainty of evidence for the results of the two subgroups—pure traumatic brain injury and mixed disease—was moderate and very low, respectively (Supplementary Table 2).
(6) Subgroup analysis was conducted on the basis of the types of control group interventions, which were divided into two subgroups: no auditory stimuli and conventional sounds. Six studies (Tavangar et al., 2015; Hoseinzadeh et al., 2017; Çevik and Namik, 2018; Mohammadi et al., 2019; Varghese et al., 2021; Xiao et al., 2023) included a control group with no auditory stimuli, with a pooled MD of 2.65 (95% CI: 1.76–3.53, *p* < 0.00001). Five studies (Parveen et al., 2015; Bahonar et al., 2019a; Yekefallah et al., 2021; Hoseini et al., 2022; Goudarzi-Rad et al., 2023) included a control group with conventional sound, with a pooled MD of 1.89 (95% CI: 1.15–2.63, *p* < 0.00001). There was no significant difference between the two subgroups (*p* = 0.20). These results indicated that auditory stimulation can significantly improve the level of consciousness of patients with DOC, regardless of whether the control group received no auditory stimulation or conventional sound stimulation (Figure 16). However, the certainty of evidence for the results of the two subgroups—no auditory stimuli and conventional sound—was very low and low, respectively (Supplementary Table 2).
(7) Subgroup analysis was conducted on the basis of intervention duration per session, and the studies were divided into three subgroups: those with intervention durations of 0–15 min per session, those with durations of 15–30 min per session, and those with durations of 30–60 min per session. Seven included studies (Parveen et al., 2015; Tavangar et al., 2015; Hoseinzadeh et al., 2017; Çevik and Namik, 2018; Mohammadi et al., 2019; Yekefallah et al., 2021; Hoseini et al., 2022) had an intervention duration of 0–15 min per session, with a pooled MD of 2.31 (95% CI: 1.70–2.92, *p* < 0.00001). Three studies (Bahonar et al., 2019a; Goudarzi-Rad et al., 2023; Xiao et al., 2023) had an intervention duration of 15–30 min per session, with a pooled MD of 1.83 (95% CI: −0.01 to 3.67; *p* = 0.05). One study (Varghese et al., 2021) had an intervention duration of 30–60 min per session, with a pooled MD of 3.6 (95% CI: 1.65–5.55, *p* = 0.0003). There was no significant difference between the three subgroups (*p* = 0.39). Significant between-group differences were observed for the 0–15 min and 30–60 min subgroups. The estimate for the 15–30 min subgroup did not meet the prespecified threshold for statistical significance (Figure 17). However, the certainty of evidence regarding the significant effects of intervention durations of 0–15 min per session and 30–60 min per session was very low (Supplementary Table 2).

**Figure 11:**
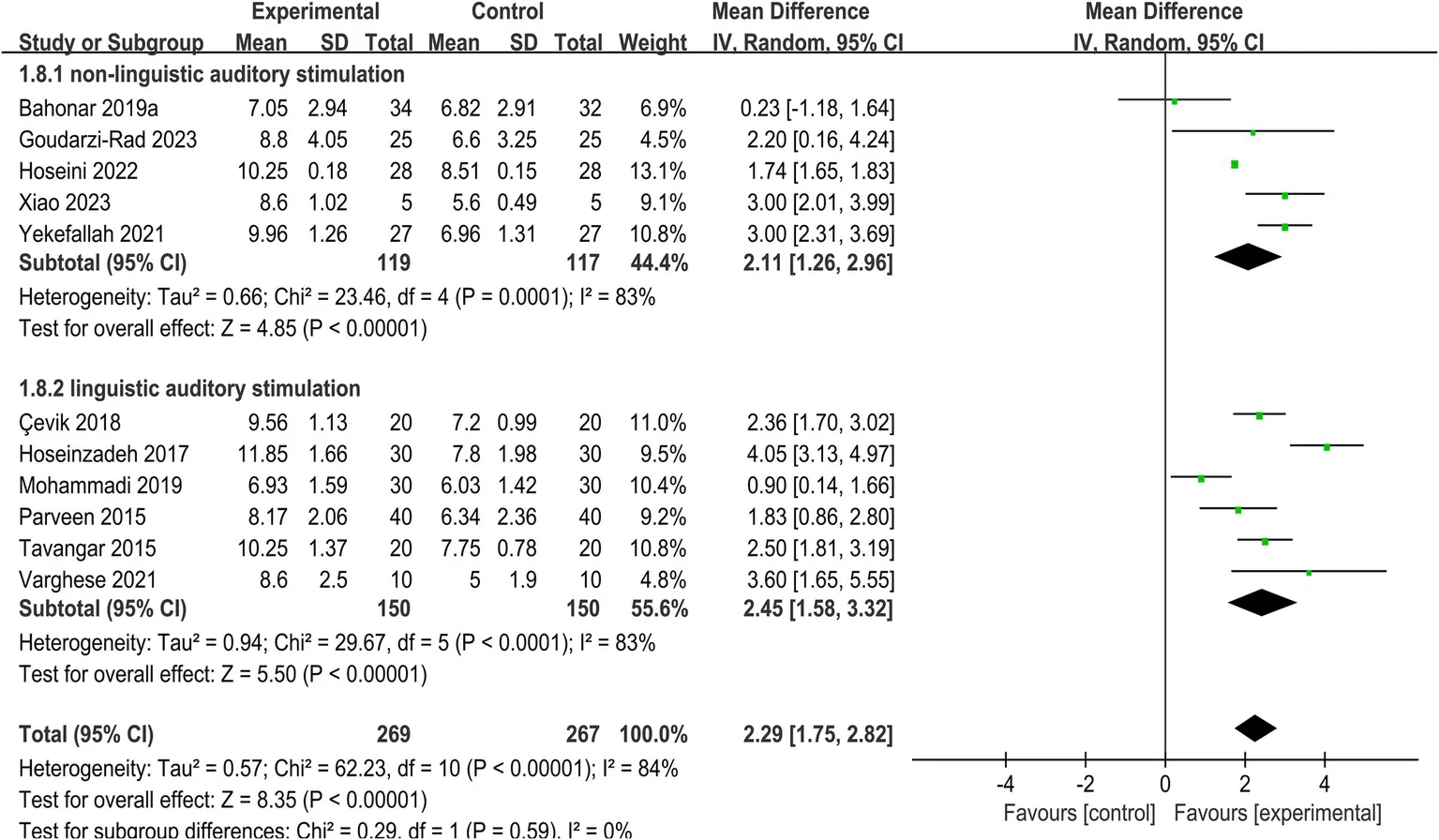
Subgroup analysis based on intervention type.

**Figure 12:**
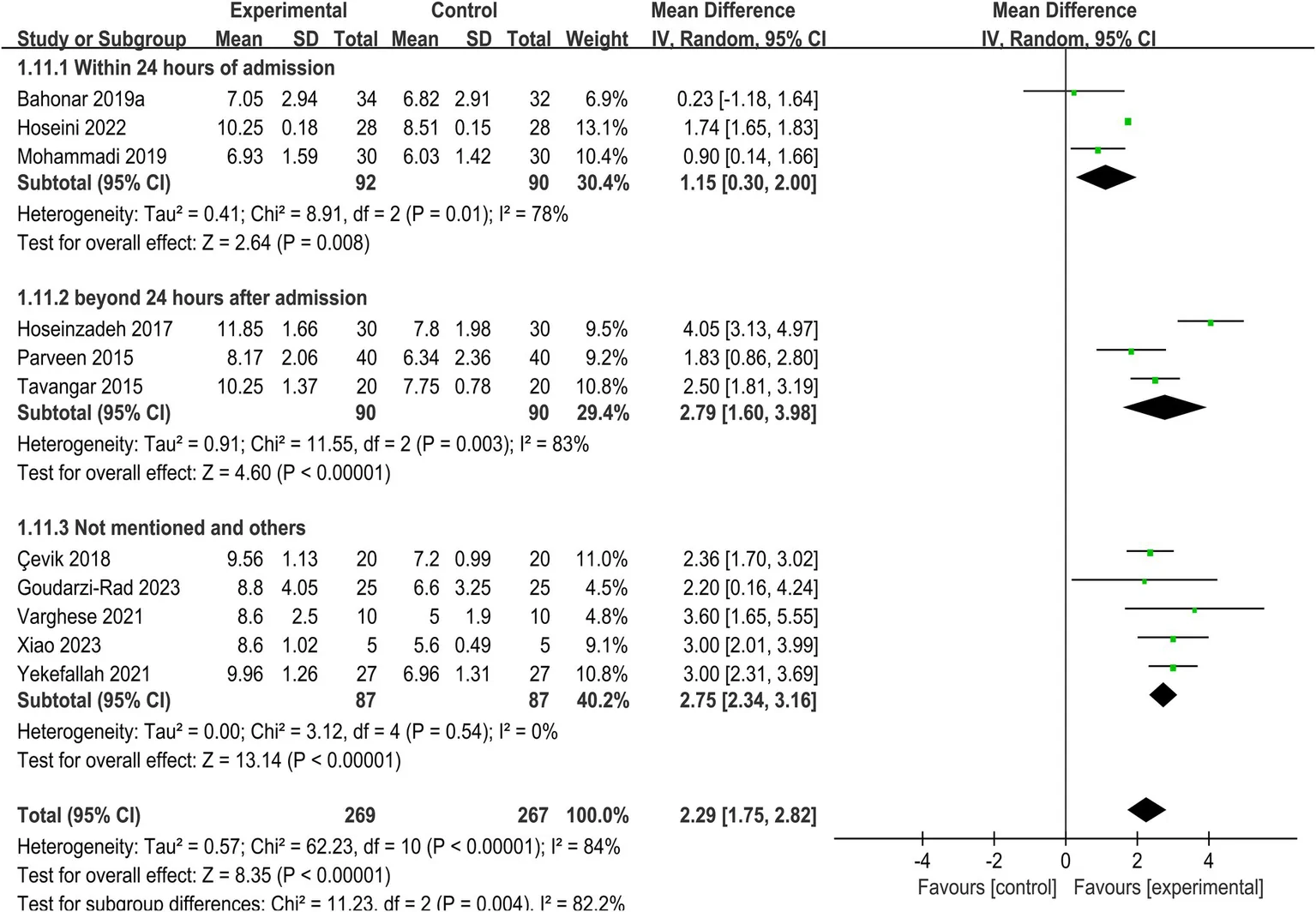
Subgroup analysis based on initiation time for GCS.

**Figure 13:**
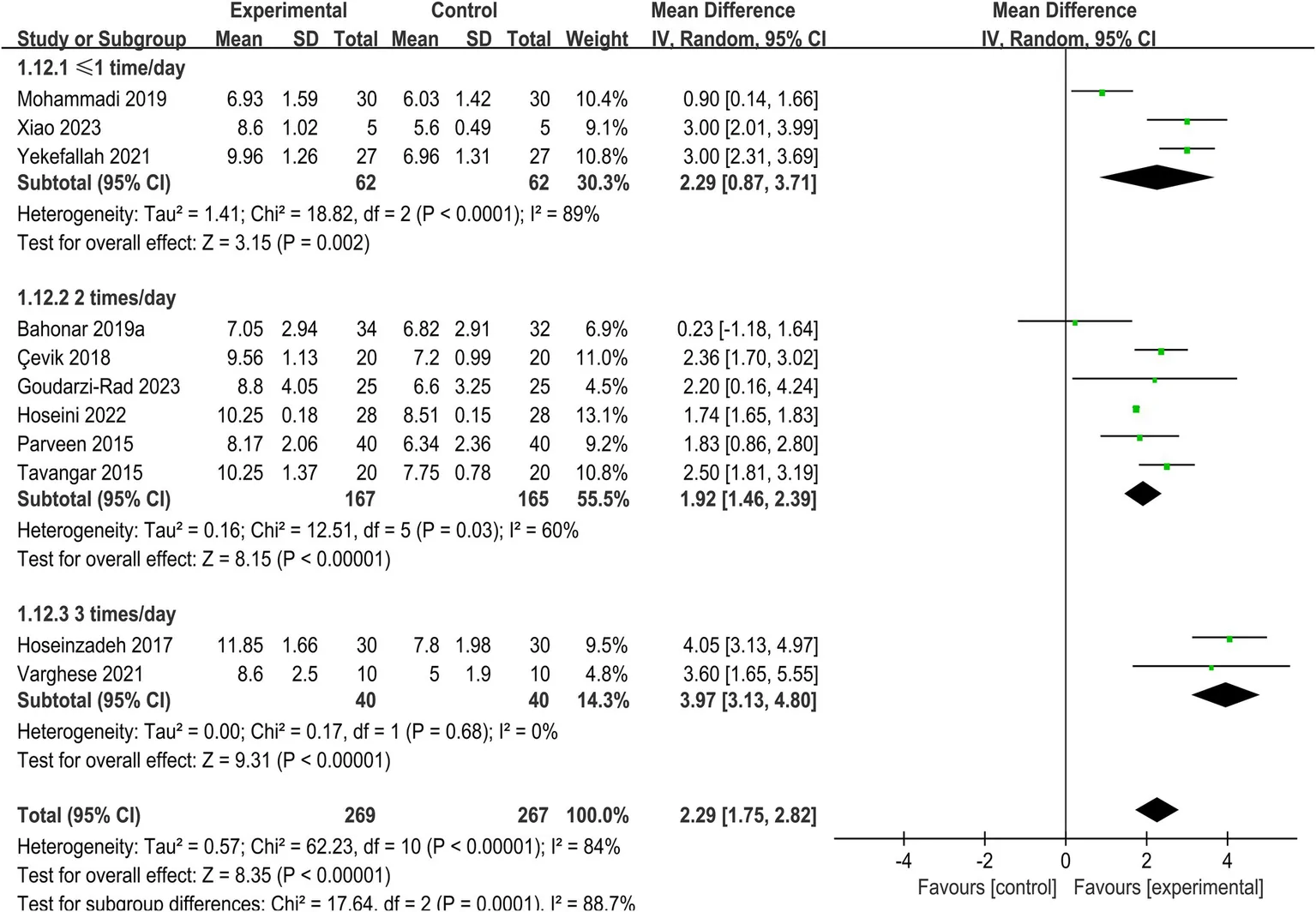
Subgroup analysis based on intervention frequency for GCS.

**Figure 14:**
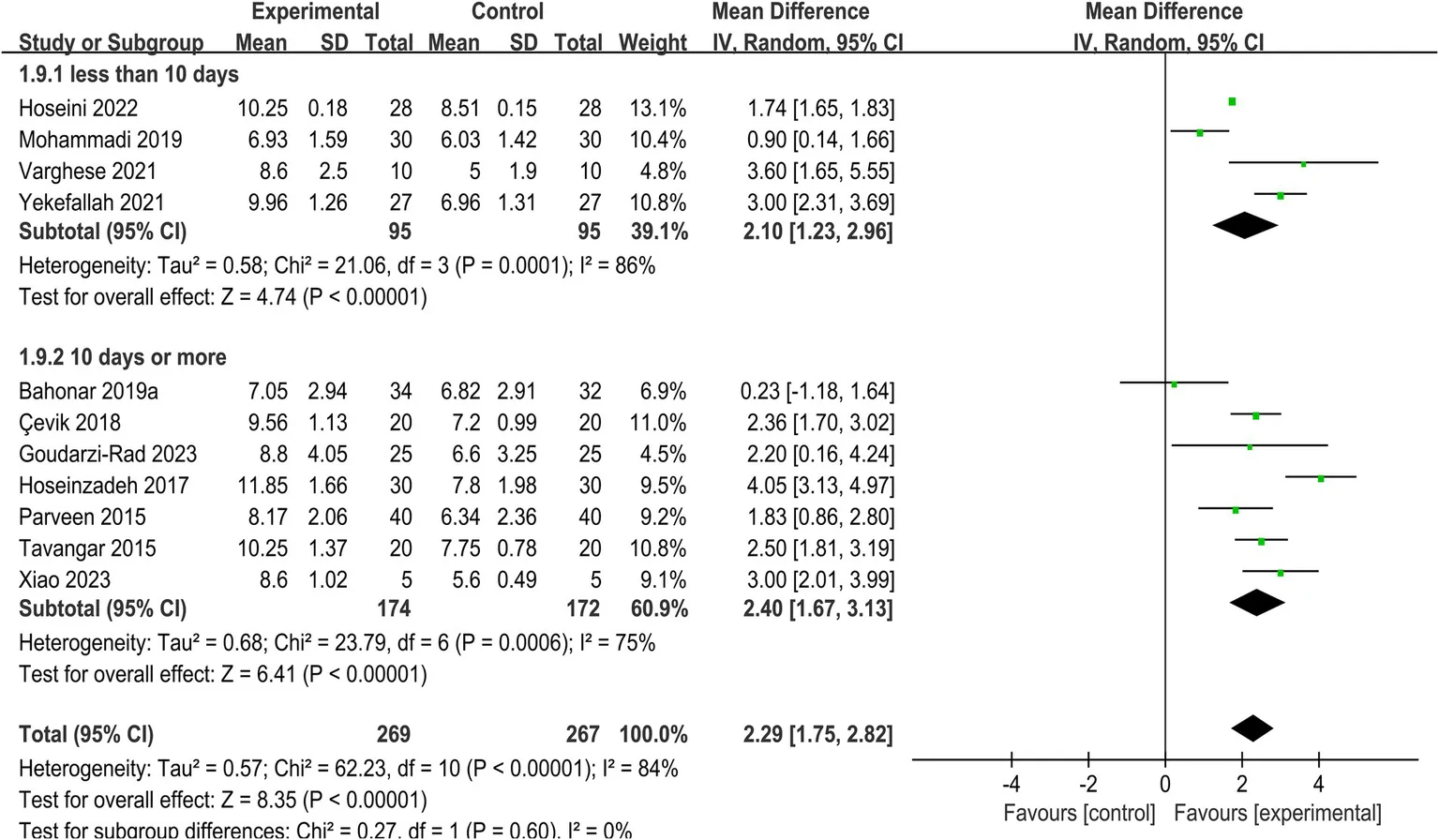
Subgroup analysis based on intervention duration for GCS.

**Figure 15:**
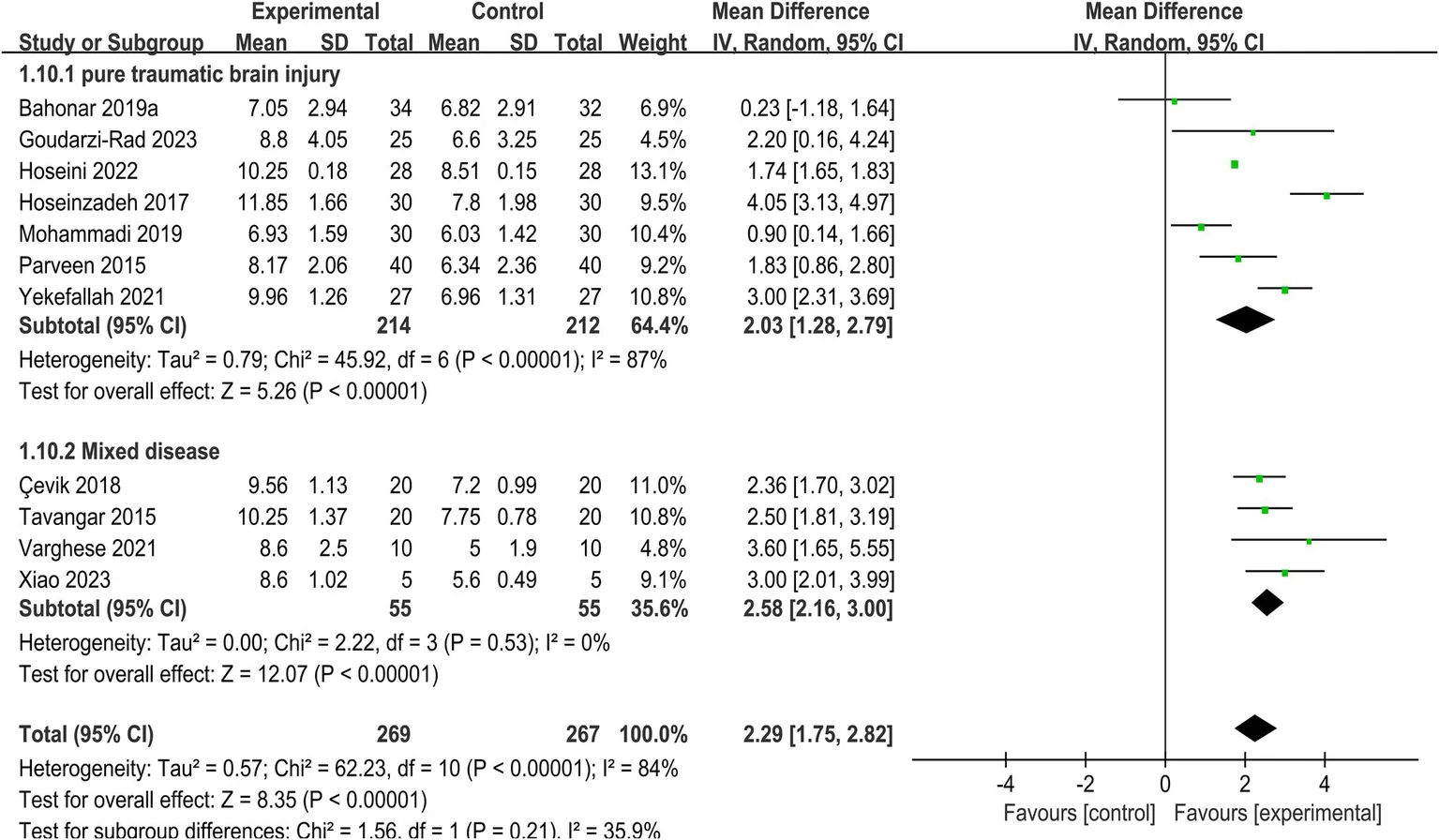
Subgroup analysis based on disease types for GCS.

**Figure 16:**
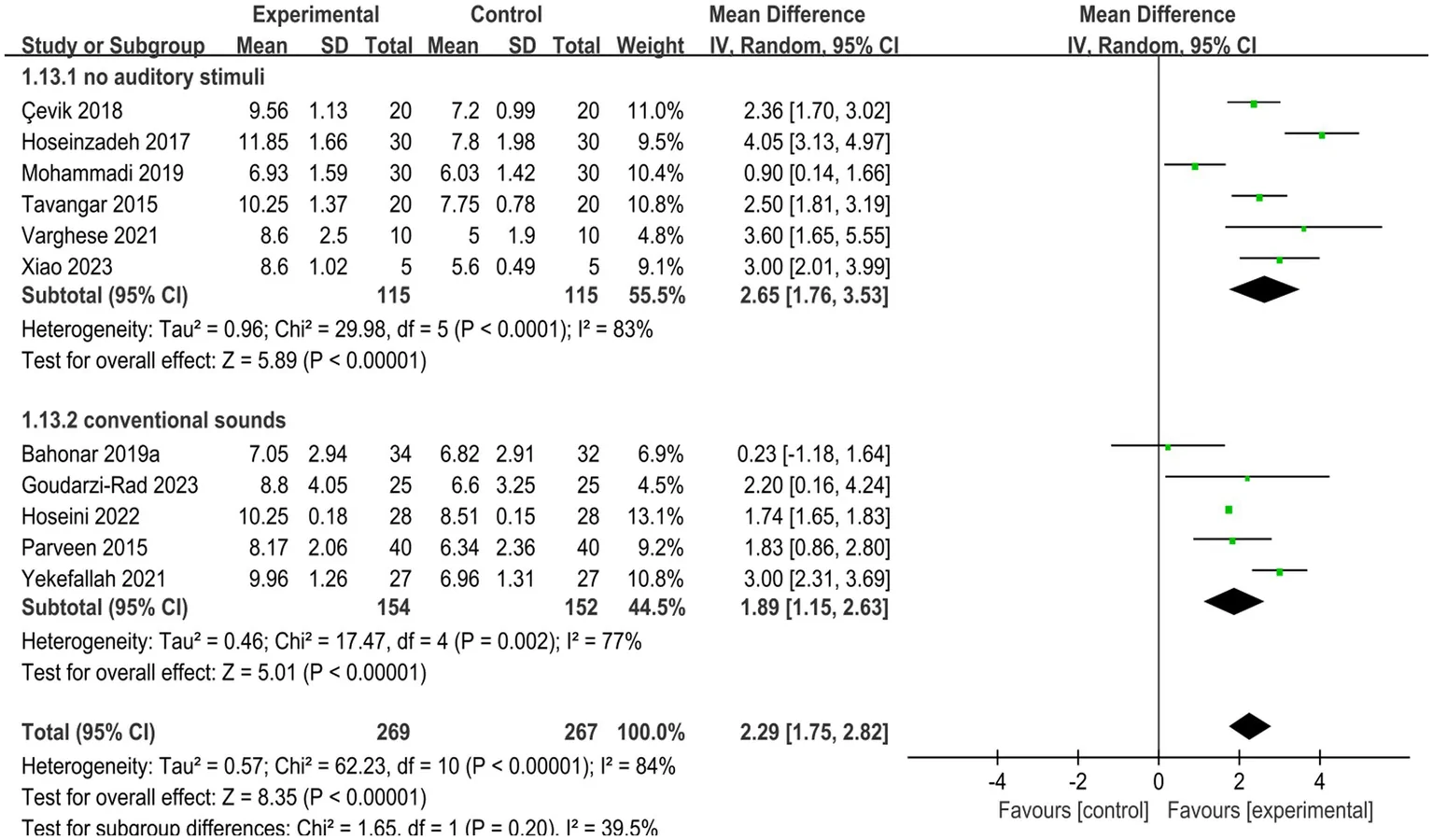
Subgroup analysis based on the types of control group interventions for GCS.

**Figure 17:**
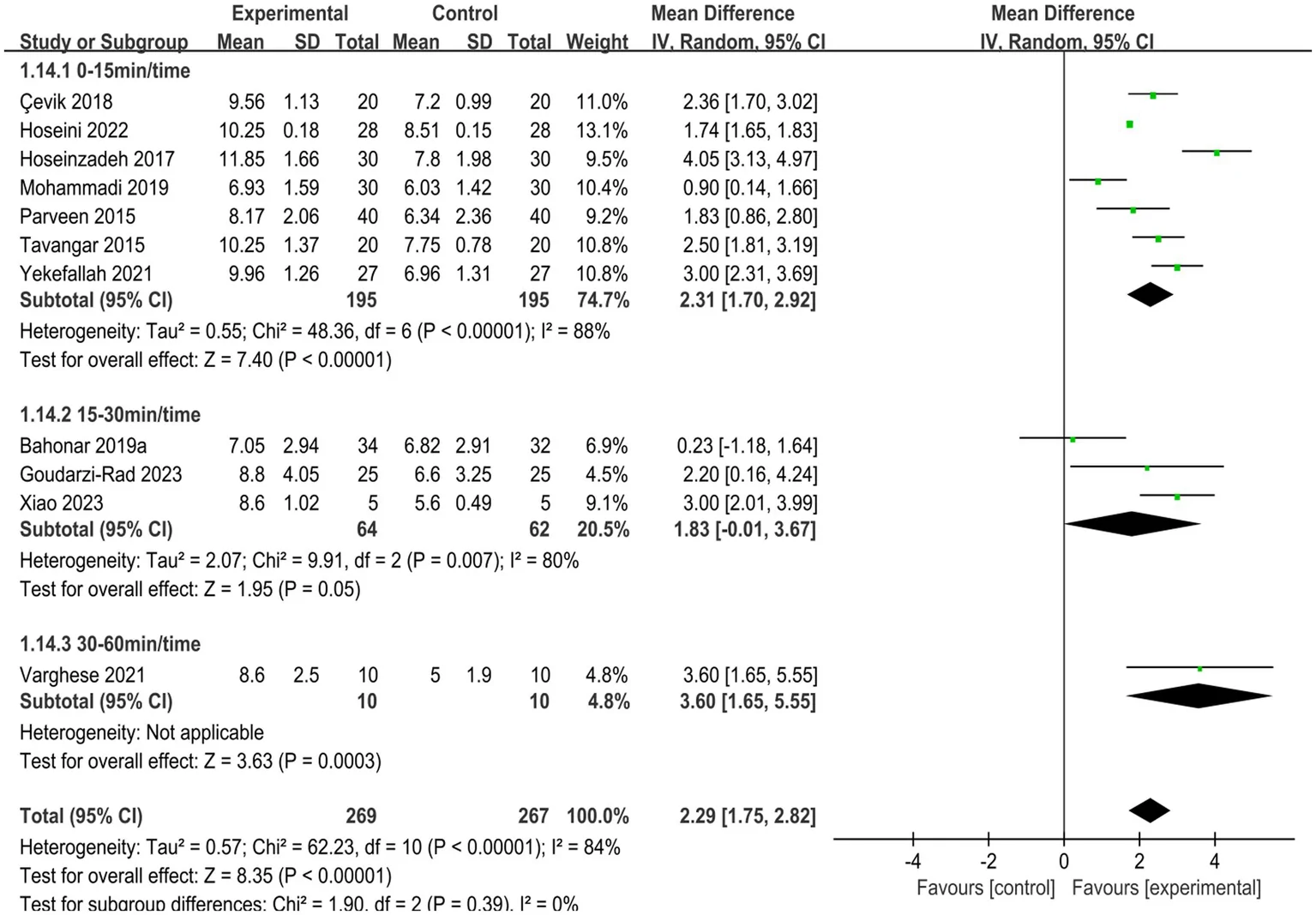
Subgroup analysis based on the intervention duration per session for GCS.

### 4.5 Results of the sensitivity analyses

The impact of individual studies on the pooled effect size was evaluated using the leave-one-out analysis. The sensitivity analysis results revealed that no single study significantly influenced the overall outcome changes in the GCS, SBP and temperature outcomes. Therefore, these three indicators were considered to exhibit relatively high robustness.

For DBP, after excluding the study by Froutan et al. (2020), the heterogeneity decreased from 49% to 0% (Figure 6), and the MD changed from −0.65 (95% CI: −2.78 to 1.49) to 1.08 (95% CI: −1.50 to 3.66); however, the findings remained nonsignificant (*p* > 0.05). For the RR, after excluding the same study (Froutan et al., 2020), the heterogeneity decreased from 89% to 0% (Figure 7), and the MD changed from −0.86 (95% CI: −5.02 to 3.29) to 1.53 (95% CI: −0.20 to 3.27); however, the findings remained nonsignificant (*p* > 0.05). For SPO_2_, after excluding the same study (Froutan et al., 2020), the heterogeneity decreased from 52% to 14% (Figure 8), and the MD changed from 0.61 (95% CI: −0.35 to 1.58) to 1.04 (95% CI: 0.21 to 1.87); furthermore, the *p-*value changed from > 0.05 to < 0.05. For the PR, after excluding the same study (Froutan et al., 2020), the heterogeneity decreased from 68% to 30% (Figure 9), and the MD changed from −9.80 (95% CI: −16.36 to −3.25) to −7.13 (95% CI: −11.51 to −2.76); the findings remained significant (*p* < 0.05). These findings indicate that the study by Froutan et al. (2020) drove the changes in statistical significance and may be the source of heterogeneity.

### 4.6 Meta-regression analysis

To explore potential sources of heterogeneity in the primary outcome (GCS score), univariable random-effects meta-regression analyses were performed. The results are presented in Supplementary Table 3. None of the examined moderators, including risk of bias, intervention type, intervention frequency, intervention duration, intervention duration per session, disease type, or control type, showed statistically significant moderating effects, with all overall *p-*values exceeding 0.05.

Substantial residual heterogeneity remained across the models, with residual *I*^2^ values ranging from 74.60% to 86.27%. Except for intervention frequency, all moderator variables had negative adjusted *R*^2^ values, suggesting that these variables did not substantially explain the between-study heterogeneity. Given the limited number of included studies (*n* = 11) and the small number of studies within some moderator categories, the findings of the meta-regression analyses should be interpreted cautiously and considered exploratory (Supplementary Table 3).

### 4.7 Publication bias

A funnel plot was examined for the GCS outcome (Supplementary Figure 1). Given the limited number of studies and substantial between-study heterogeneity, visual inspection was insufficient to exclude publication bias. Since the number of studies included in other outcome indicators and subgroup analyses was less than 10, no assessment of the risk of publication bias was conducted.

### 4.8 Certainty of evidence

According to the GRADE assessment, the certainty of evidence was low for GCS scores and very low for all physiological outcomes, including SBP, DBP, RR, SpO_2_, PR, and body temperature. The certainty of evidence for subgroup analyses ranged from very low to moderate. Outcome-specific ratings and reasons for downgrading are presented in Supplementary Table 2.

## 5 Discussion

This meta-analysis evaluated the effects of auditory SS on consciousness and physiological parameters in patients with DOC. Auditory SS was associated with higher postintervention GCS scores and a lower postintervention PR than control conditions. No statistically significant between-group differences were detected in SBP, DBP, RR, SpO_2_, or body temperature.

### 5.1 Level of consciousness for the GCS

It has been suggested that the behavioral and neural changes in patients with DOC could be mediated by postinjury environmental factors, with cognitive impairment, sensory deprivation and sensory overload contributing to poor recovery and negative neuroplasticity changes, whereas regulated sensory stimulation contributes to better recovery and blood flow in intracerebral arteries as well as positive neuroplasticity changes (Miller et al., 2013; Tomaszczyk et al., 2014; de la Tremblaye et al., 2019).

A laboratory study revealed that auditory SS can increase the levels of neurotrophic factors and dopamine, which facilitate neurogenesis and neuroplasticity and lead to improvements in attention and cognitive function (Ziabreva et al., 2003; Sliwinska-Kowalska et al., 2005; Pauwels et al., 2014). At the cellular level, exposure to frequent and various types of auditory sensory stimulation may enhance dendritic growth, stimulate a higher threshold of neurons in the reticular activating system (RAS) and improve synaptic connectivity within neural networks (NIH Consensus Development Panel, 1998; Delaware Health and Social Services, 2026; Pape et al., 2012).

From the perspective of brain structure and function, auditory sensory stimulation may reduce intracranial pressure and improve cerebral circulation and perfusion, thereby further enhancing the neuroplasticity of relevant brain regions in patients and consequently improving their level of consciousness (Park and Davis, 2016). The temporal poles, parietal lobe, PFC, cingulate, insular, and cerebellar regions are known to play a role in multiple aspects of auditory stimulus processing, including auditory semantic understanding (Xiao et al., 2023), melody and rhythm perception, emotional processing, voice recognition, and information processing of speech and tone (Platel et al., 1997; Blood et al., 1999; Thaut, 2003; Koelsch et al., 2004; Thaut et al., 2014; Paquette et al., 2017). Notably, the PFC, cingulate cortex and insula are also closely linked to the salience network (SN) and default mode network (DMN) of the brain, which have been suggested to underlie higher-level executive, attentional, and affective processes (Luo et al., 2014). Auditory SS may also affect the RAS and increase arousal and attention to incoming stimuli (Walker et al., 1998; Bassampour et al., 2008; Pape et al., 2012; Tanaka and Kudo, 2012; Gorji et al., 2014).

The results of the present meta-analysis demonstrated that postintervention GCS scores were, on average, 2.29 points higher in the auditory SS groups than in the control groups. This between-group difference was numerically larger than the pooled mean difference of 1.73 points reported in a recent meta-analysis of transcranial direct current stimulation (Ma et al., 2023). However, these comparisons should be interpreted with caution because differences in patient populations, aetiologies, intervention protocols, stimulation parameters, treatment duration, study design, and statistical methods may contribute to variations in the observed effect sizes.

In addition, comparisons with repetitive transcranial magnetic stimulation should be interpreted cautiously because the available meta-analyses primarily used the Coma Recovery Scale-Revised (CRS-R) rather than the GCS as the primary outcome measure (Yang et al., 2023). Since the GCS and CRS-R evaluate different aspects of consciousness, direct comparisons of their effect sizes are inappropriate.

These findings should not be interpreted as evidence that auditory SS is superior to other neuromodulation techniques. Rather, these findings suggest that auditory sensory stimulation may represent a promising intervention for promoting consciousness recovery in patients with disorders of consciousness. Future head-to-head randomized controlled trials using standardized intervention protocols and uniform outcome measures are warranted to directly compare the effectiveness of different neuromodulation strategies. Furthermore, according to the GRADE assessment, the certainty of evidence for the GCS outcome was rated as low. Therefore, the observed magnitude of benefit should be interpreted cautiously and requires confirmation by future high-quality randomized controlled trials.

### 5.2 Physiological parameters

Nature sounds and music can promote psychological processes and physical activities by reducing anxiety, alleviating stress, and regulating emotions, thereby providing conditions conducive to human and environmental health (Bratman et al., 2015). Nature sounds and music help patients recover from sympathetic nervous system activation caused by psychological stress and reduce sympathetic nervous activity (Chlan, 2000). The autonomic nervous system controls various bodily functions: the sympathetic branch governs primarily activation and mobilization, whereas the parasympathetic branch regulates recovery and relaxation (Andreassi, 2010). When certain types of music or sounds are played during surgical procedures, the need for sedatives and analgesics may decrease, suggesting that auditory input may modulate human responses to surgical stress (Gulledge and Kline, 1981; Cruise et al., 1997; Koch et al., 1998), thereby accelerating the recovery process. On the other hand, music has also been shown to alter the levels of intestinal hormones, endorphins, adrenaline, and catecholamines (McKinney et al., 1997; Brody, 1984). Additionally, patients with DOC are typically housed in ICUs, neurosurgery wards, and intensive rehabilitation units. Compared with other wards, environmental noises such as monitor alarms in these settings may induce fear and stress in patients, leading to elevated heart rates (Shapiro and Berland, 1972; Fontaine, 1994). Listening to music or sounds in the ward can help mask these unpleasant environmental noises. The voices of family members in auditory SS constitute linguistic auditory materials and possess unique advantages. The voices of loved ones can provide a greater sense of security through familial presence, effectively alleviating patient fear, stabilizing emotions, enhancing hopefulness, and dispelling anxiety and confusion, thereby improving PR.

Evidence that auditory SS improves PR further corroborates the mechanism by which auditory SS enhances consciousness levels. Auditory SS activates the autonomic nervous system, reduces sympathetic nerve activity in patients, and improves physiological indicators. Furthermore, according to hypothalamic–sympathetic–adrenal axis theory, this type of stimulation upwardly activates the brainstem and hypothalamus, thus enhancing functional brain regions associated with consciousness levels (Sihvonen et al., 2017). These brain regions further regulate the autonomic nervous system, thereby improving physiological indicators and forming a virtuous cycle.

As mentioned above, auditory SS can improve haemodynamic responses by modulating psychological, sympathetic nervous system, and environmental factors. However, the present meta-analysis found no statistically significant between-group differences in postintervention SBP, DBP, RR, SpO_2_, or Temp. These findings should not be interpreted as evidence of no effect. This outcome may be related to the measurement time points of the indicators in the literature included in this study. The effects of auditory SS on SBP, DBP, RR, SpO_2_ and Temp may be time sensitive. A study titled "Music Therapy as a Complementary Therapy for Mechanically Ventilated Patients" demonstrated that, in the music therapy group, patients' SBP, DBP, and RR decreased during the therapy session as well as at 5, 30, and 60 min posttherapy but slightly increased after 60 min (Almerud and Petersson, 2003). What’s more is that auditory SS may result in small, time-dependent changes in SpO_2_, with transient increases in SpO_2_ during stimulation not necessarily translating into sustained improvement. In some studies, relatively high initial SpO_2_ levels in some studies may also limit the scope for further improvement, potentially contributing to a nonsignificant pooled effect (Giesbrecht Puggina et al., 2011; Parveen et al., 2015).

The high heterogeneity in the physiological parameter indicators may be related to differences in the haemodynamic status of the trial participants, measurement time points of the evaluation indicators, types of interventions, initiation time of the interventions, frequency of the interventions, and duration of the interventions across these studies. In particular, the inclusion of the trial by Froutan et al. (2020) contributed substantially to heterogeneity in the present meta-analysis. This may be partly attributable to its assessment of outcomes 30 min after stimulation, a timing that differed considerably from that used in other studies but also may have allowed the capture of different physiological responses (Froutan et al., 2020; Parveen et al., 2015; Moghaddam et al., 2016).

Owing to the limited number of studies included for secondary outcomes, further subgroup analyses were not conducted to explore the optimal FITT protocol for improving physiological indicators or the factors contributing to heterogeneity.

### 5.3 Exploratory analyses of FITT-related intervention characteristics

The FITT principle (frequency, intensity, time, and type) of exercise provides a useful framework for designing rehabilitation interventions. Different frequencies, intensities, types, and durations of sensory stimulation may produce different therapeutic effects in patients with disorders of consciousness (DOC). Because substantial heterogeneity was observed in the pooled GCS analysis, subgroup analyses and subsequent univariable meta-regression analyses were performed to explore whether FITT-related intervention characteristics and other study-level factors could explain the observed heterogeneity.

In both the nonlinguistic and linguistic auditory SS subgroups, postintervention GCS scores were significantly higher in the intervention groups than in the control groups, with no statistically significant difference between the two subgroups. Higher postintervention GCS scores were also observed in comparisons with both no auditory stimulation and conventional auditory stimulation. For the overall intervention period, both the <10-day and ≥10-day subgroups showed statistically significant intervention–control differences, with no statistically significant subgroup difference. For session duration, postintervention GCS scores were significantly higher in the intervention groups in the 0–15 min and 30–60 min subgroups, whereas the difference in the 15–30 min subgroup did not reach the prespecified threshold for statistical significance. However, the test for subgroup differences was not statistically significant, providing insufficient evidence that treatment effects varied by session duration.

Subgroup analyses also suggested that auditory sensory stimulation initiated either within 24 h of admission or beyond 24 h after admission significantly increased the level of consciousness in patients with DOC. Postintervention GCS scores were significantly higher in the auditory SS groups than in the control groups in both initiation time subgroups (≤24 h and >24 h after admission) and across all examined intervention frequency subgroups (≤1, 2, and 3 sessions/day). The estimated between-group differences were numerically larger in the >24 h and 3-session/day subgroups. These findings are consistent with previous evidence suggesting that repeated sensory stimulation may facilitate neural plasticity and promote relearning in the injured brain (Pape et al., 2012). However, these subgroup findings should be interpreted with caution because subgroup analyses are observational by nature and may be influenced by differences in study characteristics.

To further investigate the substantial heterogeneity observed in the pooled GCS analysis, univariable random-effects meta-regression analyses were performed. None of the examined moderators, including risk of bias, intervention type, intervention frequency, intervention duration, intervention duration per session, disease type, and control type, showed statistically significant moderating effects. Furthermore, substantial residual heterogeneity remained after adjustment, suggesting that these study-level characteristics could not adequately explain the observed between-study heterogeneity. The remaining heterogeneity may be attributable to other unmeasured factors, such as differences in patient characteristics, severity of consciousness impairment, concomitant rehabilitation interventions, or methodological variations across studies. Because only 11 studies were available for meta-regression and several moderator categories included relatively few studies, these findings should be regarded as exploratory and interpreted cautiously.

From a clinical perspective, auditory sensory stimulation is a simple, noninvasive, and low-cost intervention that not only facilitates the recovery of consciousness but also may provide emotional support for patients with DOC. Occupational therapists, nurses, and family members can readily incorporate auditory sensory stimulation into routine rehabilitation practice. On the basis of the subgroup findings, auditory sensory stimulation initiated beyond 24 h after admission and delivered three times per day may be associated with greater increases in consciousness levels. However, because these observations were derived from subgroup analyses, were not confirmed by meta-regression analyses and were supported by low or very low certainty of evidence according to the GRADE assessment.The available evidence is insufficient to establish an optimal initiation time, frequency, intensity, or duration of auditory stimulation. These findings should therefore be considered hypothesis-generating rather than used to prescribe a specific FITT protocol. Future large-scale, high-quality randomized controlled trials are needed to determine the optimal timing and frequency of auditory sensory stimulation.

### 5.4 Future directions

This study also provides directions for future research. First, an increasing number of evaluation scales for DOC have been developed and demonstrated to possess favorable reliability and validity, among which the CRS-R is the gold standard for assessing consciousness levels (Zhang et al., 2019). The CRS-R holds unique value in diagnosing and differentiating various types of DOC. Concurrently, studies indicate that patients with MCS exhibit greater neurorehabilitation potential than those with VS (Bagnato, 2022). However, during our initial review of abstracts and full texts, we observed a lack of RCTs that utilized this scale for evaluation. Therefore, future research should prioritize conducting more studies employing the CRS-R for assessment while also further distinguishing the therapeutic effects of the auditory SS on different types of DOC patients. Notably, patients with disorders of consciousness exhibit substantial clinical heterogeneity. Different DOC subtypes (coma, VS/UWS, and MCS) differ considerably in terms of the degree of preserved consciousness, brain network integrity, and residual cognitive function, which may lead to differential responses to auditory sensory stimulation. Previous studies have suggested that, compared with patients in coma or VS/UWS, patients with MCS retain greater thalamocortical connectivity and higher-order cognitive network function, enabling more effective processing of external sensory stimuli. Consequently, patients with MCS may theoretically derive greater clinical benefits from auditory sensory stimulation (Edlow et al., 2021; Zhang et al., 2026). However, because most randomized controlled trials included in the present review did not report outcome data separately for different DOC subtypes, determining whether treatment effects differed across levels of consciousness was not possible. Future studies should adopt stratified study designs according to DOC subtypes (coma, VS/UWS, and MCS) and directly compare the effects of auditory sensory stimulation across different levels of consciousness. In particular, because patients with MCS generally retain relatively preserved cortical networks and greater responsiveness to external stimuli, future research should investigate whether this subgroup derives greater therapeutic benefits from auditory sensory stimulation. Second, research on the neural plasticity mechanisms underlying auditory SS-induced improvements in DOC is scarce. Future studies could delve deeper into the related neural mechanisms and conduct systematic reviews to further clarify the therapeutic mechanisms of auditory SS. Third, DOC in patients may persist long-term. However, few studies have conducted prolonged follow-up studies on DOC scores postintervention or postdischarge. Future research should consider the long-term effects of auditory SS on DOC patients to maximize consciousness recovery and provide a scientific basis for incorporating auditory SS into home rehabilitation programs for DOC patients. Fourth, although this study performed subgroup analyses on auditory SS parameters on the basis of the frequency, time, and type (FITT) principle to explore the optimal auditory SS dosage for clinical rehabilitation, the findings were limited owing to heterogeneity and certainty of evidence. Future research should further investigate FITT-based auditory SS protocols to explore tailored rehabilitation prescriptions for DOC patients, thereby providing a scientific foundation for precision rehabilitation therapy.

### 5.5 Limitations

This study has several limitations. First, substantial heterogeneity was observed for some outcomes, particularly the GCS score. To explore potential sources of heterogeneity, we performed subgroup analyses based on the FITT framework and subsequently conducted univariable random-effects meta-regression analyses. Although the subgroup analyses suggested that intervention initiation time and intervention frequency might be associated with differences in treatment effects, the meta-regression analyses did not identify any statistically significant study-level moderators. Therefore, the observed heterogeneity could not be adequately explained by the available study characteristics, and no definitive conclusions regarding the optimal FITT protocol could be drawn.

Moreover, although the FITT framework was adopted in the present study, the intensity component of auditory sensory stimulation could not be evaluated because most included trials did not report stimulation intensity (e.g., sound pressure level or volume). Consequently, subgroup analyses based on stimulation intensity were not feasible, limiting the comprehensive evaluation of the FITT framework.

The remaining heterogeneity may be attributable to other unmeasured factors, such as the stage of disease, DOC subtypes (coma, VS/UWS, and MCS), severity of consciousness impairment, baseline neurological status, concomitant rehabilitation therapies, and variations in auditory stimulation protocols. However, because these variables were inconsistently reported or unavailable in most of the included studies, further subgroup analyses or meta-regression analyses could not be performed. In addition, only 11 studies were available for the meta-regression analyses, and several moderator categories included relatively few studies, limiting the statistical power of the analyses. Therefore, the findings regarding heterogeneity should be considered exploratory and interpreted with caution.

Second, this meta-analysis was based on postintervention endpoint values rather than change-from-baseline scores. Although most included studies did not identify obvious between-group baseline imbalances for the corresponding outcomes, endpoint analyses cannot completely eliminate the potential influence of residual baseline imbalances. Furthermore, because only aggregate study-level data were available and baseline-adjusted outcome data were not consistently reported, uniform baseline-adjusted analyses could not be performed across all outcomes.

Third, the included studies were conducted in different countries and healthcare settings. Cultural, linguistic, and sociocultural differences, as well as variations in routine clinical care, may have influenced the effects of auditory sensory stimulation and contributed to the observed heterogeneity.

Fourth, relatively few studies reported physiological outcomes; therefore, subgroup analyses and meta-regression analyses were not performed for the secondary outcomes.

Fifth, publication bias could not be ruled out, as the limited number of studies and substantial between-study heterogeneity constrained the interpretation of the funnel plot.

Finally, according to the GRADE assessment, the certainty of evidence was low for GCS scores and very low for all physiological outcomes. Therefore, the pooled estimates should be interpreted cautiously. Future well-designed, adequately powered randomized controlled trials with standardized intervention protocols, comprehensive reporting of stimulation parameters (particularly stimulation intensity), and standardized outcome measures are needed to confirm these findings and establish the optimal FITT prescription for auditory sensory stimulation.

## 6 Conclusion

Our study demonstrated that auditory SS was associated with higher postintervention GCS scores and a lower postintervention PR than control conditions, suggesting that auditory SS may be a beneficial adjunctive rehabilitation intervention. However, no significant effects on SBP, DBP, RR, SpO_2_ or Temp were detected.

Subgroup analyses suggested that auditory SS initiated more than 24 h after admission and delivered three times per day was associated with greater increases in consciousness levels. However, these findings were not confirmed by subsequent meta-regression analyses and were supported by low or very low certainty of evidence. Therefore, these findings should be interpreted cautiously and should not be considered definitive clinical recommendations.

Although we explored potential sources of heterogeneity using subgroup analyses and meta-regression, no significant study-level moderators were identified. Furthermore, because most of the included studies did not report stimulation intensity (e.g., sound pressure level or volume), the intensity component of the FITT framework could not be evaluated. Variations in auditory stimulation protocols, patient characteristics, and insufficient reporting of stratified data have limited further investigations of the relationships between FITT parameters and treatment outcomes.

Finally, according to the GRADE assessment, the certainty of evidence for several outcomes was low or very low. The available evidence is insufficient to establish an optimal FITT-based protocol. Therefore, the observed beneficial effects of auditory SS should be interpreted with caution and require confirmation by future large-scale, high-quality randomized controlled trials using standardized intervention protocols and outcome measures.

## Data Availability

The original contributions presented in the study are included in the article/Supplementary material, further inquiries can be directed to the corresponding author.

## Supplementary material

SUPPLEMENTARY FIGURE 1 Funnel plot for GCS.

Supplementary Table 1 Characteristics of Included Studies.

Supplementary Table 2 GRADE certainty of evidence by outcome and subgroup.

Supplementary Table 3 Univariate Meta-regression Analyses for GCS Effect Size.

Supplementary Material 1 PROSPERO Registration Information.

Supplementary Material 2 Detailed search strategy.
