# Supplementary material for: Effects of auditory sensory stimulation on consciousness and physiological parameters among individuals with disorders of consciousness: a systematic review and meta-analysis
Source: Front Neurosci. 2026 Oct 28;20:1879145. doi: 10.3389/fnins.2026.1879145 (PMC13751540; doi:10.3389/fnins.2026.1879145)
Supplement: Supplementary file 1 [file supplementary_file_1.pdf]

NIHR | National Institute for Health and Care Research

PROSPERO
International prospective register of systematic reviews

Efficacy of Auditory Sensory Stimulation on Consciousness Levels and Physiological Parameters in Patients With Disorder of Consciousness: A Systematic Review and Meta-Analysis

Jinqin Zhang, Xinyue Yan, Fubiao Huang

Citation
Jinqin Zhang, Xinyue Yan, Fubiao Huang. Efficacy of Auditory Sensory Stimulation on Consciousness Levels and Physiological Parameters in Patients With Disorder of Consciousness: A Systematic Review and Meta-Analysis. PROSPERO 2025 CRD420251022357. Available from https://www.crd.york.ac.uk/PROSPERO/view/CRD420251022357.

REVIEW TITLE AND BASIC DETAILS

Review title

Efficacy of Auditory Sensory Stimulation on Consciousness Levels and Physiological Parameters in Patients With Disorder of Consciousness: A Systematic Review and Meta-Analysis

Condition or domain being studied

Disorders of Consciousness;Consciousness Levels ;Auditory Sensory Stimulation

Rationale for the review

Disorders of consciousness (DOC) encompass pathological alterations in states of consciousness resulting from structural/functional damage to neural networks governing arousal and awareness, including coma, vegetative state (unresponsive wakefulness syndrome, VS/UWS), and minimally conscious state (MCS) as defined by standardized diagnostic criteria. The principal objective of neurorehabilitation in patients with DOC focuses on enhancing consciousness recovery. While pharmacological agents and neuromodulatory interventions remain cornerstone therapies, non-invasive approaches such as auditory sensory stimulation have emerged as complementary therapeutic modalities. However, clinical evidence regarding their efficacy remains inconsistent. This systematic review employs meta-analytic methods to quantitatively evaluate the comparative effectiveness of auditory sensory stimulation versus conventional

rehabilitation in improving both consciousness levels and physiological parameters in DOC populations.

Review objectives

What is the comparative efficacy of auditory sensory stimulation versus conventional rehabilitation therapy in improving consciousness levels and physiological parameters in patients with DOC?

Keywords

Systematic review; Meta-analysis; Disorder of Consciousness; Auditory stimulation

Country

China

ELIGIBILITY CRITERIA

Population

Included

Participants: Individuals diagnosed with disorders of consciousness, irrespective of etiology (traumatic/non-traumatic brain injury, hypoxic-ischemic encephalopathy, etc.)

Intervention(s) or exposure(s)

Included

The experimental group received auditory sensory stimulation in addition to ordinary rehabilitation protocols administered to the control group, with no restrictions imposed on intervention modalities.

Comparator(s) or control(s)

Included

The control group was under no sound stimulus or regular environmental sound.

Study design

Only randomized study types will be included.

Included

Randomized controlled trials (RCTs) published in English, with baseline comparability confirmed between experimental and control groups (p > 0.05 for demographic/clinical characteristics)

Excluded

Non-randomized study designs, including observational studies (cross-sectional surveys, cohort analyses), case reports, narrative reviews, meta-analyses, systematic reviews, preclinical investigations and so on

Context

Inclusion Criteria

Outcomes:

1.Primary outcome: Glasgow Coma Scale (GCS) scores;

2.Secondary outcomes: Physiological parameters, including core body temperature, systolic/diastolic blood pressure, and so on.

exclusion criteria

1.Insufficient outcome data;

2.Duplicate publications;

3.Unavailable full-text articles;

4.Underpowered studies with fewer than 10 participants per treatment arm;

5.Publications in languages other than English.

TIMELINE OF THE REVIEW

Date of first submission to PROSPERO
09 April 2025

Review timeline
Start date: 8 March 2025. End date: 31 May 2025.

Date of registration in PROSPERO
09 April 2025

AVAILABILITY OF FULL PROTOCOL

Availability of full protocol
A full protocol has been written and uploaded to PROSPERO. The protocol will be made available after the review is completed.

SEARCHING AND SCREENING

Search for unpublished studies
Only published studies will be sought.

Main bibliographic databases that will be searched
The main databases to be searched are *Embase.com*, *PubMed* and *Scopus*.

Other important or specialist databases that will be searched
Cochrane; web of science;ProQuest

Search language restrictions
The review will only include studies published in English.

Search date restrictions
Databases will be searched for articles published before 8 March 2025, there are no restrictions on search start date.

Other methods of identifying studies
No other methods will be used.

Addiitonal information about identifying studies

searching references of other related META analysis articles.

Link to search strategy

A full search strategy has been uploaded to PROSPERO. The PDF may be accessed through this link https://www.crd.york.ac.uk/PROSPEROFILES/e95aaf99404b9829e1058824c6ebe3e6.pdf.

Selection process

Studies will be screened independently by at least two people (or person/machine combination) with a process to resolve differences.

Other relevant information about searching and screening

None

DATA COLLECTION PROCESS

Data extraction from published articles and reports

Data will be extracted independently by at least two people (or person/machine combination) with a process to resolve differences.

Authors will be asked to provide any required data not available in published reports.

Study risk of bias or quality assessment

Risk of bias will be assessed using: *Cochrane RoB-2*

Data will be assessed independently by at least two people (or person/machine combination) with a process to resolve differences.

Additional information will be sought from study investigators if required information is unclear or unavailable in the study publications/reports.

Reporting bias assessment

Risk of bias due to missing results will be assessed

Certainty assessment

The certainty of the evidence was assessed independently by two reviewers using the Grading of Recommendations, Assessment, Development and Evaluation (GRADE) method (Guyatt et al., 2008). Since only RCT studies were included in this review five downgrading factors were considered: risk of bias, inconsistency, indirectness, imprecision and publication bias. The strength of the evidence was rated as "high," "moderate," "low," or "very low." Any differences were resolved by the third researcher.

OUTCOMES TO BE ANALYSED

Main outcomes

Primary outcome:

Glasgow Coma Scale (GCS) scores;

Definition:

The Glasgow Coma Scale (GCS) is a standardized tool used to assess a person's level of consciousness, particularly after head injury or in critical care. It evaluates three components: eye opening, verbal response, and motor response, with total scores ranging from 3 (deep unconsciousness) to 15 (fully alert).

Time point(s) at which measured:

Before and after auditory sensory stimulation intervention.

Measures of effect：

Mean Difference (MD) = Mean of experimental group - Mean of control group

Additional outcomes

Secondary outcomes:

Physiological parameters, including core body temperature, systolic/diastolic blood pressure, and so on.

Time point(s) at which measured:

Before and after auditory sensory stimulation intervention.

Measures of effect：

Mean Difference (MD) = Mean of experimental group - Mean of control group

PLANNED DATA SYNTHESIS

Strategy for data synthesis

Statistical analysis was conducted using Review Manager 5.4 or Stata 18 software, employing appropriate statistical methods. Continuous variables were reported as weighted mean differences (MD) with 95% confidence intervals (CI); standardized mean differences (SMD) were applied when units varied substantially across studies.Heterogeneity was assessed quantitatively using the I^2 statistic. If heterogeneity was low ($I^2 \leqslant 50\%$, P ⩾ 0.1), a fixed-effects model was used for meta-analysis. If significant heterogeneity existed (I^2 > 50%, P < 0.1), potential sources were explored through subgroup analyses and sensitivity analyses. When no obvious clinical heterogeneity was present or inter-study differences remained statistically nonsignificant, a random-effects model was applied.Statistical significance was determined at P < 0.05 in the meta-analysis difference test.

CURRENT REVIEW STAGE

Stage of the review at this submission

Review stage	Started	Completed
Pilot work	✓	✓
Formal searching/study identification	✓	✓
Screening search results against inclusion criteria	✓	✓
Data extraction or receipt of IPD		

Review stage	Started	Completed
Risk of bias/quality assessment		
Data synthesis		

Review status
The review is currently planned or ongoing.

Publication of review results
Results of the review will be published in English.

REVIEW AFFILIATION, FUNDING AND PEER REVIEW

Review team members
Miss Jinqin Zhang (review guarantor and contact) ORCID: 0000-0002-1313-8936. Faculty of Rehabilitation, Capital Medical University;china rehabilitation research center. China.

No conflict of interest declared.

Miss Xinyue Yan. 1. Rehabilitation Therapeutics, School of Nursing, Jilin University;Faculty of Rehabilitation, Shandong University of Traditional Chinese Medicine. China.

No conflict of interest declared.

Dr Fubiao Huang. Faculty of Rehabilitation, Capital Medical University;Department of Occupational Therapy, China Rehabilitation Research Center. China.

No conflict of interest declared.

Named contact
Miss Jinqin Zhang (m13051384533@163.com). ORCID: 0000-0002-1313-8936. Faculty of Rehabilitation, Capital Medical University;china rehabilitation research center. China.

Review affiliation
main organisational affiliation:

1. Faculty of Rehabilitation, Capital Medical University ;2. Department of Occupational Therapy, China Rehabilitation Research Center.

Funding source

Grant number
2023ZX-Q11

Additional non-commercial funding information
China Rehabilitation Research Center Youth fund project

Grant number(s) State the funder, grant or award number and the date of award

2023ZX-Q11

21-7-2023

Additional commercial funding information
This META analysis has no commercial funders.

Peer review

There has been no peer review of this planned review.

ADDITIONAL INFORMATION

Review conflict of interest

Declared individual interests are recorded under team member details. This review is funded by a commercial organisation.. No additional interests are recorded for this review.

Medical Subject Headings

Acoustic Stimulation; Consciousness Disorders; Humans; Medicine; Meta-Analysis as Topic; Systematic Reviews as Topic

SIMILAR REVIEWS

Check for similar records already in PROSPERO

PROSPERO identified a number of existing PROSPERO records that were similar to this one (last check made on 30 March 2025). These are shown below along with the reasons given by that the review team for the reviews being different and/or proceeding.

- The Effects of Integrated Sensory Stimulation On Consciousness Levels and Cognitive Functions in Patients Requiring Intensive Care Units' Stay: A Systematic Review [published 10 October 2024] [CRD42024594210]. The review was judged **not to be similar**
- Efficacy and safety of transcranial magnetic stimulation for disorders of consciousness: a systematic review and meta-analysis [published 9 September 2022] [CRD42022351957]. The review was judged **not to be similar**
- Systematic Review of Tactile-Based Interventions Combined with Multisensory Stimulation Protocols in the Rehabilitation of Patients with Disorders of Consciousness [published 26 March 2025] [CRD420251013057]. The review was judged **not to be similar**

PROSPERO version history

- Version 1.0, published 09 Apr 2025

Disclaimer

The content of this record displays the information provided by the review team. PROSPERO does not peer review registration records or endorse their content.

PROSPERO accepts and posts the information provided in good faith; responsibility for record content rests with the review team. The guarantor for this record has affirmed that the information provided is truthful and that they understand that deliberate provision of inaccurate information may be construed as scientific misconduct.

PROSPERO does not accept any liability for the content provided in this record or for its use. Readers use the information provided in this record at their own risk.

Any enquiries about the record should be referred to the named review contact

Protocol

The systematic review and meta-analysis were performed in accordance with PRISMA guidelines and Cochrane Handbook recommendations, following the PICOS (Population, Interventions, Comparisons, Outcomes, and Study design) framework.

1. Data Sources and Search Strategy

In accordance with systematic review and meta-analysis guidelines, we comprehensively searched PubMed, Embase, Cochrane Library, Web of Science, Scopus, and Ovid databases. The search was restricted to English-language publications up to 8 March, 2025. We employed a combination of controlled vocabulary (MeSH terms for PubMed, Emtree for Embase) and free-text keywords, with core search terms including *Consciousness Disorder*, *Acoustic Stimulation*, and *Randomized Controlled Trials*.

To ensure literature saturation, we additionally:

- Screened reference lists of relevant articles and review papers
- Conducted manual searches of key journals
- Performed supplementary searches using Google Scholar to identify additional eligible studies

This multi-source approach minimized the risk of missing pertinent studies.

2. Inclusion and Exclusion Criteria

2.1 Inclusion Criteria

1. **Study design:** Only randomized controlled trials (RCTs) were eligible. Baseline characteristics must have demonstrated comparability between intervention and control groups. Studies were restricted to English-language publications.
2. **Participants:** Patients with any etiology of impaired consciousness.
3. **Interventions:** The experimental group received acoustic stimulation (any protocol) in addition to standard care, while the control group received either no sound intervention or routine environmental sound exposure.
4. **Outcomes:**
    - **Primary outcome:** Glasgow Coma Scale (GCS) scores

- **Secondary outcomes:** Physiological parameters including core temperature, systolic/diastolic blood pressure, oxygen saturation, heart rate, and respiratory rate

2.2 Exclusion Criteria

Studies were excluded if they:

1. Lacked RCT design (e.g., surveys, descriptive studies, meta-analyses, systematic reviews, preclinical research, or study protocols)
2. Provided insufficient data for quantitative analysis
3. Duplicate publications
4. Unavailable as full-text articles
5. Had fewer than 10 participants (small sample size threshold)
6. Were published in languages other than English

3 Literature Screening and Data Extraction

1) The literature retrieved from each database was imported into the literature management software NoteExpress and uniformly screened. Two researchers independently conducted preliminary screening based on the inclusion and exclusion criteria, removing literature that did not meet the requirements. They then read the titles and abstracts, removing content that was clearly irrelevant, and downloaded the full text of articles that might meet the inclusion criteria for this study. The researchers decided whether to include the articles and extracted the literature independently. After both researchers had extracted the relevant data, they cross-checked their results. If discrepancies arose, they were resolved through discussion or by consulting a third party to determine whether the literature should be included. 2) Two researchers extracted data from the included articles, inputting the data into an Excel table created in advance. The extracted data included general information (first author, publication time, country, research type, sample size), patient characteristics (sample size, proportion of males and females, age distribution, disease type, baseline characteristics), intervention methods (intervention methods and specific details for the control and trial groups), and outcome indicators (Glasgow Coma Scale, body

temperature, systolic blood pressure, diastolic blood pressure, etc.).

4 Quality assessment

Each included RCTs quality was evaluated by two researchers using The Cochrane Collaboration's tool. The evaluation tool was consisted of seven items assessing the random sequence generation, the allocation concealment, the blinding of participants and personnel, the blinding of outcome assessment, the incomplete outcome data, the selective reporting and other bias. The quality of each item was rated as low risk, high risk, or unclear and was indicated by three difference colors. If there was a disagreement between the two researchers on the results, the third researcher with judging ability was decided.

5 Certainty of evidence

The certainty of the evidence was assessed independently by two reviewers using the Grading of Recommendations, Assessment, Development and Evaluation (GRADE) method (Guyatt et al., 2008). Since only RCT studies were included in this review five downgrading factors were considered: risk of bias, inconsistency, indirectness, imprecision and publication bias. The strength of the evidence was rated as "high," "moderate," "low," or "very low." Any differences were resolved by the third researcher.

6 Statistical analysis

Data analysis was performed using ReviewManager 5.4 and STATA 18.0 software, and statistical analysis was conducted according to relevant statistical methods. Continuous variables were expressed as 95% confidence intervals (95% CI), mean values, and weighted mean differences (mean difference, MD). When there were large differences between units in the research data, standardized mean differences (standardized mean difference, SMD) were used. The formulas included: $Mean_{change}$ formula: $Mean_{change} = Mean_{post} - Mean_{pre}$, the SD_{change}formula: SD_{change} = root square $(SD_{pre}^2 + SD_{post}^2) – 2 * correlation * SD_{pre} * SD_{post}$

Combining I^2 to quantitatively judge the degree of heterogeneity, if I^2

<25%, it indicates that there is low heterogeneity between the study results, if I^2 is between 25%-50%, it indicates that there is moderate heterogeneity between the study results, and if I^2 >50%, it indicates that there is high heterogeneity between the study results. When heterogeneity is low, the fixed effect model is used to combine the effect size, and when heterogeneity is high, the random effect model is used for meta-analysis. Subgroup analysis will be conducted according to age, sex, baseline level of consciousness disorder, disease type, intervention time frequency, etc. to evaluate the source of heterogeneity, and a one-to-one sensitivity analysis will be used to evaluate whether a single trial can drive the results. The difference test P value <0.05 of the meta-analysis results will be used as the criterion for judging that the difference between the two groups has statistical significance. If the meta-analysis comparison includes more than 10 studies, the potential publication bias will be analyzed through a funnel plot or Egger's test.
