# Supplementary material for: Effects of auditory sensory stimulation on consciousness and physiological parameters among individuals with disorders of consciousness: a systematic review and meta-analysis
Source: Front Neurosci. 2026 Oct 28;20:1879145. doi: 10.3389/fnins.2026.1879145 (PMC13751540; doi:10.3389/fnins.2026.1879145)
Supplement: Supplementary file 2 [file supplementary_file_2.pdf]

Supplementary Material 2 . Detailed search strategy

Database	Search strategy	Laster update	Results
PubMed	(((((((((((((((((((("Consciousness Disorders"[Mesh]) OR (Consciousness Disorder[Title])) OR (Disorder of Consciousness[Title])) OR (Disorders of Consciousness[Title])) OR (Consciousness, Level Depressed[Title])) OR (Depressed Level of Consciousness [Title])) OR (Consciousness, Level Altered[Title])) OR (Altered Level of Consciousness[Title])) OR (Semiconsciousness[Title])) OR (altered state of consciousness[Title])) OR (hypotonic hyporesponsive episode[Title])) OR (lethargy[Title])) OR (minimally conscious state[Title])) OR (unconsciousness[Title])) OR (Vegetative state[Title])) OR (Impaired consciousness[Title])) OR (disorders of consciousness [Title])) OR (Disturbance of consciousness[Title])) OR (Coma[Title])) OR (Unreponsive wakefulness syndrome[Title])) AND ((("Acoustic Stimulation"[Mesh]) OR (Auditory Stimuli[Title/Abstract])) OR (Auditory Stimulation[Title/Abstract])) OR (Auditory sensory stimulation[Title/Abstract])) OR (Stimulation, Auditory[Title/Abstract])) OR (Stimulation, Acoustic[Title/Abstract])) OR (Auditory sensory[Title/Abstract])) OR (Evoked Potentials, Auditory[Title/Abstract])) OR (Sound[Title/Abstract])) OR (Voice[Title/Abstract])) OR (Voices[Title/Abstract])) OR (Vocal[Title/Abstract])) OR (sounds[Title/Abstract])) OR (Music[Title/Abstract])) OR (Call[Title/Abstract])) OR (Calling[Title/Abstract])) OR (call-name[Title/Abstract])) OR (Language[Title/Abstract])) OR (TV[Title/Abstract])) OR (Radio[Title/Abstract])) OR (Recordings[Title/Abstract])) OR (Loudspeakers[Title/Abstract])) OR (Noice[Title/Abstract])) OR (Noises[Title/Abstract])) OR (Pure tone stimulation[Title/Abstract])) OR (Tale[Title/Abstract])) OR (Story[Title/Abstract])) OR (speech[Title/Abstract])) OR (Storytelling[Title/Abstract])) OR (Speaking[Title/Abstract])) OR (Reading[Title/Abstract])) OR (Headphones[Title/Abstract]))) OR (earphones[Title/Abstract])) OR (Auditory Evoked Potentials[Title/Abstract])) OR (Clapping[Title/Abstract])) OR (Bells[Title/Abstract])) OR (blocks[Title/Abstract])) OR (clap[Title/Abstract])) OR (Tuning fork[Title/Abstract])) OR (bell[Title/Abstract])) OR (Tone Burs	April 25, 2026	74

	ts[Title/Abstract])) OR (standard tones[Title/Abstract])) OR (deviant tones[Title/Abstract])) OR (novel stimuli[Title/Abstract])) OR (passive auditory oddball paradigm[Title/Abstract])) OR (clicks[Title/Abstract])) OR (Non-verbal sounds[Title/Abstract]))) AND ((((Randomized controlled trials[Title/Abstract]) OR (Randomized[Title/Abstract])) OR (Clinical trial[Title/Abstract])) OR (placebo[Title/Abstract]))		
Embase	#1. 'Consciousness Disorder':ab,ti or 'Disorder of Consciousness':ab,ti or 'Disorders of Consciousness ':ab,ti or 'Depressed Level of Consciousness':ab,ti or 'Consciousness, Level Depressed':ab,ti or 'Consciousness, Level Altered':ab,ti or 'Altered Level of Consciousness':ab,ti or 'Semiconsciousness':ab,ti or 'altered state of consciousness':ab,ti or 'hypotonic hyporesponsive episode':ab,ti or 'lethargy':ab,ti or 'unconsciousness':ab,ti or 'minimally conscious state':ab,ti or 'Vegetative state':ab,ti or 'Impaired consciousness ':ab,ti or 'disorders of consciousness':ab,ti or 'Disturbance of consciousness':ab,ti or 'Coma':ab,ti or 'Unreponsive wakefulness syndrome':ab,ti 95213<br>#2. 'Acoustic Stimulation':ab,ti or 'Auditory Stimulation':ab,ti or 'Auditory Stimuli':ab,ti or 'Auditory sensory stimulation':ab,ti or 'Stimulation, Auditory':ab,ti or 'Stimulation, Acoustic':ab,ti or 'Auditory sensory':ab,ti or 'Evoked Potentials, Auditory':ab,ti or 'Sound':ab,ti or 'Voice':ab,ti or 'Voices':ab,ti or 'Vocal ':ab,ti or 'sounds':ab,ti or 'Music':ab,ti or 'Call':ab,ti or 'Calling':ab,ti or 'call-name':ab,ti or 'Language':ab,ti or 'TV':ab,ti or 'Radio':ab,ti or 'Recordings':ab,ti or 'Loudspeakers':ab,ti or 'Loudspeakers':ab,ti or 'Noice':ab,ti or 'Noises':ab,ti or 'Pure tone stimulation':ab,ti or 'Tale':ab,ti or 'Story':ab,ti or 'speech':ab,ti or 'Storytelling':ab,ti or 'Speaking':ab,ti or 'Reading':ab,ti or 'Headphones':ab,ti or 'earphones':ab,ti or 'Auditory Evoked Potentials':ab,ti or 'Clapping':ab,ti or 'Bells':ab,ti or 'blocks':ab,ti or 'clap':ab,ti or 'Tuning fork':ab,ti or 'bell':ab,ti or 'Tone Bursts':ab,ti or 'standard tones':ab,ti or 'deviant tones':ab,ti or 'novel stimuli':ab,ti or 'passive auditory oddball paradigm':ab,ti or 'clicks':ab,ti or 'Non-verbal sounds':ab,ti 1628415<br>#3. 'Randomized controlled trials':ab,ti or 'Randomi	April 25, 2026	451

	zed':ab,ti or 'placebo':ab,ti or 'Clinical trial':ab,ti 1825990<br>#4 #1 AND #2 AND #3 451		
Cochrane	#1 (Consciousness Disorder):ti,ab,kw or (Disorder of Consciousness):ti,ab,kw or (Disorders of Consc iousness):ti,ab,kw or (Consciousness, Level Depres sed):ti,ab,kw or (Depressed Level of Consciousnes s):ti,ab,kw or (Consciousness, Level Altered):ti,ab,k w or (Altered Level of Consciousness):ti,ab,kw or (Semiconsciousness):ti,ab,kw or (altered state of c onsciousness):ti,ab,kw or (hypotonic hyporesponsive episode):ti,ab,kw or (lethargy):ti,ab,kw or (minima lly conscious state):ti,ab,kw or (unconsciousness):ti, ab,kw or (Vegetative state):ti,ab,kw or (Impaired c onsciousness):ti,ab,kw or (disorders of consciousn ess):ti,ab,kw or (Disturbance of consciousness):ti,a b,kw or (Coma):ti,ab,kw or (Unreponsive wakefuln ess syndrome):ti,ab,kw 9440<br>#2 (Auditory Stimulation):ti,ab,kw or (Auditory S timuli):ti,ab,kw or (Auditory sensory stimulation):ti, ab,kw or (Stimulation, Auditory):ti,ab,kw or (Stimu lation, Acoustic):ti,ab,kw or (Auditory sensory):ti,a b,kw or (Evoked Potentials, Auditory):ti,ab,kw or (Sound):ti,ab,kw or (Voice):ti,ab,kw or (Voices):ti,a b,kw or (Vocal):ti,ab,kw or (sounds):ti,ab,kw or (Music):ti,ab,kw or (Call):ti,ab,kw or (Calling):ti,a b,kw or (call-name):ti,ab,kw or (Language):ti,ab,kw or (TV):ti,ab,kw or (Radio):ti,ab,kw or (Recordin gs):ti,ab,kw or (Loudspeakers):ti,ab,kw or (Noice):t i,ab,kw or (Noises):ti,ab,kw or (Pure tone stimulati on):ti,ab,kw or (Tale):ti,ab,kw or (Story):ti,ab,kw o r (speech):ti,ab,kw or (Storytelling):ti,ab,kw or (Sp eaking):ti,ab,kw or (Reading):ti,ab,kw or (Headpho nes):ti,ab,kw or (earphones):ti,ab,kw or (Auditory Evoked Potentials):ti,ab,kw or (Clapping):ti,ab,kw o r (Bells):ti,ab,kw or (blocks):ti,ab,kw or (clap):ti,a b,kw or (Tuning fork):ti,ab,kw or (bell):ti,ab,kw or (Tone Bursts):ti,ab,kw or (standard tones):ti,ab,kw or (deviant tones):ti,ab,kw or (novel stimuli):ti,ab, kw or (passive auditory oddball paradigm):ti,ab,kw or (clicks):ti,ab,kw or (Non-verbal sounds):ti,ab,k w 127663<br>#3 (Randomized controlled trials):ti,ab,kw or (Ra ndomized):ti,ab,kw or (placebo):ti,ab,kw or (Clinica	April 25, 2026	736

	l trial):ti,ab,kw 1519589<br>#4 #1 and #2 and #3 736		
Scopus	(TITLE-ABS-KEY ("Consciousness Disorder" OR "Consciousness Disorders" OR "Disorder of Consciousness" OR "Disorders of Consciousness" OR "Consciousness, Level Depressed" OR "Depressed Level of Consciousness" OR "Consciousness, Level Altered" OR "Altered Level of Consciousness" OR "Semiconsciousness" OR "altered state of consciousness" OR "hypotonic hyporesponsive episode" OR "lethargy" OR "minimally conscious state" OR "unconsciousness" OR "Vegetative state" OR "Impaired consciousness " OR "disorders of consciousness" OR "Disturbance of consciousness" OR "Coma" OR "Unreponsive wakefulness syndrome") AND TITLE-ABS-KEY ("Acoustic Stimulation" OR "Auditory Stimulation" OR "Auditory Stimuli" OR "Auditory sensory stimulation" OR "Stimulation, Auditory" OR "Stimulation, Acoustic" OR "Auditory sensory" OR "Evoked Potentials, Auditory" OR "Sound" OR "Voice" OR "Voices" OR "Vocal " OR "sounds" OR "Music" OR "Call" OR "Calling" OR "call-name" OR "Language" OR "TV" OR "Radio" OR "Recordings" OR "Loudspeakers" OR "Noice" OR "Noises" OR "Pure tone stimulation" OR "Tale" OR "Story" OR "speech" OR "Storytelling" OR "Speaking" OR "Reading" OR "Headphones" OR "earphones" OR "Auditory Evoked Potentials" OR "Clapping" OR "Bells" OR "blocks " OR "clap" OR "Tuning fork" OR "bell" OR "Tone Bursts" OR "standard tones" OR "deviant tones" OR "novel stimuli" OR "passive auditory oddball paradigm" OR "clicks" OR "Non-verbal sounds") AND TITLE-ABS-KEY ("Randomized controlled trials" OR "Randomized" OR "placebo" OR "Clinical trial"))	April 25, 2026	1823
Web of science	Search:TS=(Consciousness Disorder) OR TS=(Consciousness Disorders) OR TS=(Disorder of Consciousness) OR TS=(Disorders of Consciousness) OR TS=(Consciousness, Level Depressed) OR TS=(Depressed Level of Consciousness) OR TS=(Consciousness, Level Altered) OR TS=(Altered Level of Consciousness) OR TS=(Semiconsciousness) OR TS=(altered state of consciousness) OR TS=(hypoto	April 25, 2026	734

	nic hyporesponsive episode) OR TS=(lethargy) OR TS=(minimally conscious state) OR TS=(unconsci ousness) OR TS=(Vegetative state) OR TS=(Impair ed consciousness) OR TS=(disorders of conscious ness) OR TS=(Disturbance of consciousness) OR TS=(Coma) OR TS=(Unreponsive wakefulness syn drome) Results: 90513<br>Search: (Acoustic Stimulation) OR TS=(Auditory S timulation) OR TS=(Auditory Stimuli) OR TS=(Au ditory sensory stimulation) OR TS=(Stimulation, A uditory) OR TS=(Stimulation, Acoustic) OR TS=(Auditory sensory) OR TS=(Evoked Potentials, Au ditory) OR TS=(Sound) OR TS=(Voice) OR TS=(Voices) OR TS=(Vocal) OR TS=(sounds) OR T S=(Music) OR TS=(Call) OR TS=(Calling) OR T S=(call-name) OR TS=(Language) OR TS=(TV) O R TS=(Radio) OR TS=(Recordings) OR TS=(Loud speakers) OR TS=(Noice) OR TS=(Noises) OR TS =(Pure tone stimulation) OR TS=(Tale) OR TS=(S tory) OR TS=(speech) OR TS=(Storytelling) OR T S=(Speaking) OR TS=(Reading) OR TS=(Headpho nes) OR TS=(earphones) OR TS=(Auditory Evoke d Potentials) OR TS=(Clapping) OR TS=(Bells) O R TS=(blocks) OR TS=(clap) OR TS=(Tuning for k) OR TS=(bell) OR TS=(Tone Bursts) OR TS=(s tandard tones) OR TS=(deviant tones) OR TS=(no vel stimuli) OR TS=(passive auditory oddball para digm) OR TS=(clicks) OR TS=(Non-verbal sound s) Results: 7911922<br>Search: (Randomized controlled trials) OR TS=(Ra ndomized) OR TS=(placebo) OR TS=(Clinical tria l) Results: 2070600<br>Search: #1 AND #2 AND #3 Results: 734		
Proquest	#1 AB,TI("Consciousness Disorder" OR "Consciou sness Disorders" OR "Disorder of Consciousness" OR "Disorders of Consciousness" OR "Consciousn ess, Level Depressed" OR "Depressed Level of C onsciousness" OR "Consciousness, Level Altered" OR "Altered Level of Consciousness" OR "Semico nsciousness" OR "altered state of consciousness" OR "hypotonic hyporesponsive episode" OR "letha rgy" OR "minimally conscious state" OR "unconsc iousness" OR "Vegetative state" OR "Impaired co nsciousness " OR "disorders of consciousness" OR	April 25, 2026	285

	"Disturbance of consciousness" OR "Coma" OR "Unreponsive wakefulness syndrome") 116580<br>#2 AB,TI("Acoustic Stimulation" OR "Auditory Stimulation" OR "Auditory Stimuli" OR "Auditory sensory stimulation" OR "Stimulation, Auditory" OR "Stimulation, Acoustic" OR "Auditory sensory" OR "Evoked Potentials, Auditory" OR "Sound" OR "Voice" OR "Voices" OR "Vocal " OR "sounds" OR "Music" OR "Call" OR "Calling" OR "call-name" OR "Language" OR "TV" OR "Radio" OR " Recordings" OR "Loudspeakers" OR "Noice" OR "Noises" OR "Pure tone stimulation" OR "Tale" OR "Story" OR "speech" OR "Storytelling" OR "Speaking" OR "Reading" OR "Headphones" OR "ear phones" OR "Auditory Evoked Potentials" OR "Clapping" OR "Bells" OR "blocks" OR "clap" OR " Tuning fork" OR "bell" OR "Tone Bursts" OR "standard tones" OR "deviant tones" OR "novel stimuli" OR "passive auditory oddball paradigm" OR "clicks" OR "Non-verbal sounds") 9628669<br>#3 AB,TI("Randomized controlled trials" OR "Randomized" OR "placebo" OR "Clinical trial") 1915743<br>#4 #1 and #2 and #3 285		
