# Supplementary material for: Effects of auditory sensory stimulation on consciousness and physiological parameters among individuals with disorders of consciousness: a systematic review and meta-analysis
Source: Front Neurosci. 2026 Oct 28;20:1879145. doi: 10.3389/fnins.2026.1879145 (PMC13751540; doi:10.3389/fnins.2026.1879145)
Supplement: Supplementary file 3 [file table_1.pdf]

Supplementary Table 2. GRADE certainty of evidence by outcome and subgroup

Comparisons	Groups（No. of study）	N (comparisons)	N of patients Auditory SS	RS	MD (95%CI)	I^2(%)	p-value	Study design	Risk of bias	Inconsistency	Indirectness	Imprecision	Other considerations	Certainty[f] (GRADE)
Overall analysis														
Auditory SS vs. RS in GCS(1-11)	NA	11	265	265	2.29<br>1.75~2.82	84	<0.00001	RCT	serious [a]	serious [c]	none	none	none	⊕⊕○○<br>LOW
Auditory SS vs. RS in SBP（10,12-15）	NA	5	131	128	-4.47<br>-10.51~1.57	72	0.15	RCT	very serious [b]	seriou [c]	none	sevious [e]	none	⊕○○○<br>VERY LOW
Auditory SS vs. RS in DBP（10,12-15）	NA	5	131	128	-0.65<br>-2.78~1.49	49	0.55	RCT	very serious [b]	seriou [c]	none	serious [e]	none	⊕○○○<br>VERY LOW
Auditory SS vs. RS in RR（10,12,14,15）	NA	4	101	98	-0.86<br>-5.02~3.29	89	0.68	RCT	very serious [b]	very seriou [d]	none	serious [e]	none	⊕○○○<br>VERY LOW
Auditory SS vs. RS in SPO_2（10,12,14,15）	NA	4	101	98	0.61<br>-0.35~1.58	52	0.21	RCT	very serious [b]	seriou [c]	none	serious [e]	none	⊕○○○<br>VERY LOW
Auditory SS vs. RS in PR（10,12-15）	NA	5	131	128	-9.80<br>-16.36~--3.25	68	0.003	RCT	very serious [b]	seriou [c]	none	none	none	⊕○○○<br>VERY LOW
Auditory SS vs. RS in Temp（12,14,15）	NA	3	61	58	-0.17<br>-0.51~0.18	75	0.34	RCT	very serious [b]	seriou [c]	none	serious [e]	none	⊕○○○<br>VERY LOW
Subgroup analysis														
Auditory SS vs. RS in GCS	Intervention Type													
	non-linguistic auditory stimulation（1，2，3，5，6）	5	98	98	2.11<br>1.26~2.96	83	<0.00001	RCT	not serious	very seriou [d]	none	very serious [f]	none	⊕○○○<br>VERY LOW
	linguistic auditory	6	167	167	2.45	83	<0.00001	RCT	very	very seriou d	none	none	none	⊕○○○

	stimulation（4,7-11）				1.58~3.32				serious b					VERY LOW
Auditory SS vs. RS in GCS	Initiation Time of the Intervention													
	within 24 hours of admission （3，6，7）	3	88	88	1.15<br>0.30~2.00	78	0.008	RCT	not serious	very serious [d]	none	serious [e]	none	⊕○○○<br>VERY LOW
	beyond 24 hours of admission（9，10，11）	3	80	80	2.79<br>1.60~3.99	83	<0.00001	RCT	very serious [b]	very serious [d]	none	serious [e]	none	⊕○○○<br>VERY LOW
	not mentioned and others（1，2，4，5，8）	5	97	97	2.75<br>2.34~3.16	0	<0.00001	RCT	not serious	none	none	serious [e]	none	⊕⊕⊕○<br>MODERATE
Auditory SS vs. RS in GCS	Intervention Frequency													
	≤1 time/day（1，4，7）	3	62	62	2.29<br>0.87~3.71	89	0.002	RCT	not serious	very seriou [c]	none	serious [e]	none	⊕○○○<br>VERY LOW
	2 times/day（2，3，6，8，10，11）	6	173	173	1.92<br>1.46~2.39	60	<0.00001	RCT	serious [a]	serious	none	none	none	⊕○○○<br>VERY LOW
	3 times/day（5，9）	2	30	30	3.97<br>3.13~4.80	0	<0.00001	RCT	serious [a]	none	none	serious [e]	none	⊕⊕○○<br>LOW
Auditory SS vs. RS in GCS	Duration of Intervention													
	<10 days（3，4，5，7）	4	92	92	2.1<br>1.23~2.96	86	0.00001	RCT	serious [a]	very serious [d]	none	serious [e]	none	⊕○○○<br>VERY LOW
	≥10 days（1，2，6，8，9,10,11）	7	173	173	2.4<br>1.67~3.13	75	<0.00001	RCT	serious [a]	seriou [c]	none	none	none	⊕⊕○○<br>LOW
Auditory SS vs. RS in GCS	Disease Type													
	pure traumatic brain	7	200	200	2.03	87	<0.00001	RCT	not	seriou [c]	none	none	none	⊕⊕⊕○

	injury（2,3,4,6,7,9,10）				1.28~2.79				serious					MODERATE
	mixed disease（1,5,8,11）	4	65	65	2.58<br>2.16~3.00	0	<0.00001	RCT	very serious [b]	none	none	serious [e]	none	⊕○○○<br>VERY LOW
Auditory SS vs. RS in GCS	Types of Control Group Interventions													
	no auditory stimuli（1,5,7,8,9,11）	6	115	115	2.65<br>1.76~3.53	83	<0.00001	RCT	very serious [b]	very serious [d]	none	none	none	⊕○○○<br>VERY LOW
	conventional sound（2,3,4,6,10）	5	150	150	1.89<br>1.15~2.63	77	<0.00001	RCT	not serious	very serious [d]	none	none	none	⊕⊕○○<br>LOW
Auditory SS vs. RS in GCS	Intervention Duration per Session													
	0-15 minutes per session（3,4,7,8,9,10,11）	7	195	195	2.31<br>1.70~2.92	88	<0.00001	RCT	very serious [b]	very serious [d]	none	none	none	⊕○○○<br>VERY LOW
	15-30 minutes per session（1,2,6）	3	60	60	1.83<br>-0.01~3.67	80	0.05	RCT	not serious	very serious [d]	none	serious [e]	none	⊕○○○<br>VERY LOW
	30-60 minutes per session（5）	1	10	10	3.6<br>1.65~5.55	/	0.0003	RCT	very serious [b]	none	none	serious [e]	none	⊕○○○<br>VERY LOW

Auditory SS=auditory sensory stimulation

RS=regular stimulation

N=Number

MD=Mean Deviation

CI =Confidence Interval

GCS=Glasgow Coma Scale

SBP=Systolic Blood Pressure

DBP=Diastolic Blood Pressure

RR=Respiratory Rate

SpO_2=Peripheral Oxygen Saturation

PR=Pulse Rate

Temp=Temperature

NA=Not Applicable

RCT=Randomized Controlled Trial

a Some studies were at high risk of bias.

b Most studies were at high risk of bias.

c Downgraded by one level for serious inconsistency due to unexplained between-study heterogeneity, considering variation in effect estimates, overlap of confidence intervals, and heterogeneity statistics.

d Downgraded by two levels for very serious inconsistency due to pronounced unexplained between-study heterogeneity that substantially limited interpretation of the pooled effect.

e Imprecision due to small sample size or wide confidence intervals.

f Serious imprecision due to very small sample size and confidence intervals crossing the line of no effect.
