# Supplementary material for: Effects of auditory sensory stimulation on consciousness and physiological parameters among individuals with disorders of consciousness: a systematic review and meta-analysis
Source: Front Neurosci. 2026 Oct 28;20:1879145. doi: 10.3389/fnins.2026.1879145 (PMC13751540; doi:10.3389/fnins.2026.1879145)
Supplement: Supplementary file 4 [file table_2.pdf]

## Supplementary Table 1. Characteristics of Included Studies

| No. | First Author (Year) | Nation | Type of Research | No. of Centers | Gender （M/F） | Age | Disease Type | Level of Consciousness | Intervention of Trail Group<br>i:initiation time of the intervention<br>ii:intervention type<br>iii intervention frequency<br>iv:duration of intervention<br>v:intervention duration per session | Intervention of Control Group | Outcomes | Results | PEDro |
|---|---|---|---|---|---|---|---|---|---|---|---|---|---|
| 1 | Xiao 2023 | China | RCT | Single | T:2/3<br>C:4/1 | T:<br>26.8±11.21<br>C:<br>38.8±15.07 | MCS | NI | i:After admission<br>ii:Live music therapy by music therapist<br>iii:5times/week<br>iv:4weeks<br>v:30min/time | No auditory sensory stimulation | GCS | The results reveal that GCS (p = 0.0071**)in the music group were significantly improved compared with the control groups. | 7/10 |
| 2 | Goudarzi-Rad 2023 | Iran | RCT | Single | T:19/6<br>C:21/4 | T:<br>35.92±14.68<br>C:<br>33.32±13.74 | TBI | GCS:<br>T:5.12±1.90<br>C:5.28±1.81 | i:Random allocation after admission<br>ii:occupational sounds recorded by patients' families in their workplaces<br>iii:2times/day<br>iv:10days<br>v:15-20min/time | Auditory stimuli with the sounds of the ICU (normal sensory stimuli, staff voices, device alarms, a companion’s voice during visits, etc.). | GCS | The Mean±SD scores of GCS after the stimulation were 6.60±3.25 in the control group and 8.80±4.05 in the intervention group. This difference was statistically significant (P=0.038). The level of consciousness in the intervention group increased more than in the control group in the 10th day of study | 8/10 |
| 3 | Hoseini | Iran | RCT | Single | T:18/10 | T: | TBI | GCS: | i:Within 24h of admission | An audio file | GCS | There was a significant increase in | 7/10 |

| | | | | | | | | | | | | | |
|---|---|---|---|---|---|---|---|---|---|---|---|---|---|
| | 2022 | | | | C:23/5 | 42.46±2.42<br>C:<br>40.32±2.75 | | T:7.35±0.17<br>C:7.21±0.2 | ii:Familiar voices.First, the selected family member should introduce himself/herself, clarify the time and place, and briefly describe what happened to the patient . In the second part, which lasted four minutes, they were asked to recall and talk about positive memories, such as pleasant family gatherings or trips.<br>iii:2times/day<br>iv:6days<br>v:15min/time | that only included the familiar sounds of the ICU | | the level of consciousness on the sixth days after the intervention among the patients in the intervention group compared to the control group (P=0.001). | |
| 4 | Yekefallah 2021 | Iran | RCT | Two | T:23/4<br>C:22/5 | T:<br>36.00±13.37<br>C:<br>39.51±13.65 | HT:Head trauma with subdural, epidural, intracerebral, intraventricular, or subarachnoid hemorrhage | GCS:5-8 | i:NI<br>ii:Musical stimulation:“Beach walk” music from the “Top-Hits zum Entspannen” album (Volume 1)<br>iii:1time/day<br>iv:7days<br>v:15min/time | No musical stimulation | GCS | There were significant between-group differences respecting the posttest mean score of GCS in the seventh days of the study intervention (P < 0.05), the posttest mean score of GCS in the intervention group significantly increased in the intervention group (P < 0.0001), while it did not significantly change in the control group (P >0.05) | 7/10 |
| 5 | Varghese | India | RCT | Single | T:6/4 | 20-80 | Coma:Cer | GCS: | i:NI | The participants in | GCS | The results showed a significant | 7/10 |

| | | | | | | | | | | | | | |
|---|---|---|---|---|---|---|---|---|---|---|---|---|---|
| | 2021 | | | | C:6/4 | | ebrovascular disorders, Crainiocerebral injuries,Neuroinfections | T:6.1 ±1.197<br>C:5.6 ±1.776 | ii:Familiar voices.The Kin of the participant's sound: any event or any moment that they have spent together. Patients should be informed where they are and what is happening to them and; Should tell Patients a message of affection with an optimistic perspective, saying something about their family life.<br>iii:3times/day<br>iv:5days<br>v:60min/time | the control group were not provided with the intervention, but headphones were placed over the external pinnae of the comatose patients after covering it with the disposable and stretchable paper cloth headphone covers as a placebo. | | improvement in the level of consciousness in the interventional group compared to the control group (p = 0.003). | |
| 6 | Bahonar 2019a | Iran | RCT | Two | T:26/4<br>C:26/4 | NI | TBI | GCS:<br>T:5.00 ±1.12<br>C:4.60±1.54 | i:Within 24h of admission<br>ii:Nature sounds:A collection of night, wind, rain, waterfall, birds, river, and crickets' sounds<br>iii:2times/day<br>iv:2weeks<br>v:30min/time | Only routine care | GCS | The patients'level of consciousness was assessed using the Glasgow Coma<br>Scale before and 2 weeks after the intervention. The groups had no significant differences in the mean scores of the consciousness level before, and the last day of the intervention. Also, there was a significant difference in the number of patients who regained full | 7/10 |

| | | | | | | | | | | | | | |
|---|---|---|---|---|---|---|---|---|---|---|---|---|---|
| | | | | | | | | | | | | consciousness (P = .001) in the intervention groups compared with the control group. | |
| 7 | Mohammadi 2019 | Iran | RCT | Single | T:23/7<br>C:23/7 | T: 35.16±14.1<br>C: 38.13±13.89 | HT of any cause, Comatose state | GCS:<br>T:6.1±1.26<br>C:5.93±1.33 | i：Within the 24hours of admission<br>ii: Familiar voices:The first part of the message was included the information about time and place (thirty seconds) and the accident which had lead to head trauma (thirty seconds). In the second part that lasted four minutes, they talked about shared sweet memories. In the third part, they spoke promising and encouraging words about the patient's recovery and future subjects (five minutes).<br>iii:1time/day<br>iv:3days<br>v:10min/time | No auditory sensory stimulation | GCS | A significant increase was found in the mean GCS in the intervention group after there days auditory stimulation (P<0.05). However, no significant changes were observed in the control group (P>0.05). T | 9/10 |
| 8 | Çevik 2018 | Turkey | RCT | Single | T:17/13<br>C:16/14 | T：68.66±7.03<br>C:69.76±10.05 | DOC：Cause of respiratory system diseases，cancer ，trauma，infection ，digestive | GCS:<br>T:4.76 ± 0.85<br>C:4.70 ± 0.79 | i：Start immediately after enrollment<br>ii:Voice of a nurse:Time, place, words of encouragement<br>iii:2times/day<br>iv:10days<br>v:5-10min/time | No auditory sensory stimulation | GCS | The difference in mean GCS scores of the 2 groups was statistically significant after the inventention (P<0 .05). | 7/10 |

| | | | | | | | | | | | | | |
|---|---|---|---|---|---|---|---|---|---|---|---|---|---|
| | | | | | | | system diseases, neurology system diseases, circulatory system diseases, heart diseases and kidney diseases | | | | | | |
| 9 | Hoseinzadeh 2017 | Iran | RCT | Single | T:20/0<br>C:20/0 | 15-60 | HI | GCS:<br>T:6.05±0.75<br>C:5.90±0.64 | i：NI<br>iiVoice of a nurse:place, time and date , patient identification factors , details of family members, and some occupational information.<br>iii:3times/day<br>iv:10days<br>v:10min/time | No auditory sensory stimulation | GCS | The level of consciousness increased in both intervention and control groups, but the increase in the intervention group was more than the control group ($P < 0.01$). A significant difference in GCS between the intervention and control groups was observed on day 10 ($P < 0.001$). Repeated measures ANOVA showed significant differences within groups including time effect, interaction of time and intervention ($P < 0.001$). | 8/10 |
| 10 | Parveen | India | RCT | Single | T:34/6 | T: | TBI | GCS： | i：Within the 72 hours of admission | Routine care | GCS、 | On day 14, GCS was again | 7/10 |

| | | | | | | | | | | | | | |
|---|---|---|---|---|---|---|---|---|---|---|---|---|---|
| | 2015 | | | | C:33/7 | 34.95±13.98<br>C:<br>41.32±12.83 | | T：5.12 ± 1.20<br>C:5.10 ± 1.37 | ii:Familiar voices:close family members;calling the name of the patient, to introduce themselves and talk to the patient based on the prewritten script in such a way that it brings hope and a feeling of worthiness in the patients.Repetitive use of patient’s name was emphasized while speaking. An assurance is given to the patient that he/ she is not alone and there are many people around to help.<br>iii:2 times/day<br>iv:2weeks<br>v:10min/time | | SBP、DBP、RR、SPO2、PR | significantly higher (8.17±2.06) in intervention group as compared with (6.34±2.36) control group.<br>There was significant increase in PR and SpO2 during and after intervention. | |
| 11 | Tavangar 2015 | Iran | RCT | Single | T:16/4<br>C:16/4 | T:<br>38.05±7.43<br>C:<br>34.80±5.91 | Comatose patients with brain injury due to acute subdural hematoma | GCS:<br>T:6.25 ± 1.25<br>C:6.30 ± 1.03 | i:Patient has stable hemodynamic symptoms for at least 24 hours<br>ii:Famliar voices:family members<br>iii:2times/day<br>iv:10days<br>v:5-15min/time | No auditory sensory stimulation | GCS | On the first day before the intervention, there was no a statistically significant difference between the mean of GCS in both groups(P = 0.89), but on the tenth day after the intervention, there was a significant difference (P = 0.0001) between the mean GCS in both control and intervention groups. | 8/10 |
| 12 | Froutan | Iran | RCT | Single | T:18/10 | T: | TBI | GCS： | i:Within 24h of admission | An audio file | SBP、 | The results indicated a significant | 8/10 |

| | | | | | | | | | | | | | |
|---|---|---|---|---|---|---|---|---|---|---|---|---|---|
| | 2020 | | | | C:23/5 | 42.46±2.42<br>C:<br>40.32±2.75 | | T：7.4±1 | ii:Familiar voices.First, the selected family member should introduce himself/herself, clarify the time and place, and briefly describe what happened to the patient . In the second part, which lasted four minutes, they were asked to recall and talk about positive memories, such as pleasant family gatherings or trips. | that only included the familiar sounds of the ICU | DBP、RR、SPO2、PR、Temp | decrease in systolic blood pressure, diastolic blood pressure, respiratory rate and heart rate for the patients in the intervention group as compared to the patients in the control group (P<0.0001). Nevertheless, no significant difference was observed in temperature and oxygen saturation (P>0.05). | |
| | | | | | | | | | iii:2times/day | | | | |
| | | | | | | | | C：7.2±1.1 | iv:6days | | | | |
| | | | | | | | | | v:15min/time | | | | |
| 13 | Bahonar 2019b | Iran | RCT | Two | T:26/4<br>C::26/4 | 15-75 | TBI | GCS：<8 | i：Within the 24hours of admission | Routine Care | SBP、DBP、PR | The nature sounds group showed differences in the pulse rate from the control (p=0.015) groups 30 min after the interventions in the second day. | 8/10 |
| | | | | | | | | | ii:Nature sounds:ea collection of night, wind, rain, waterfall, birds, river, and crickets’ sounds | | | | |
| | | | | | | | | | iii:2times/day | | | | |
| | | | | | | | | | iv:2days | | | | |
| | | | | | | | | | v:30min/time | | | | |
| 14 | Moghaddam 2016 | Iran | RCT | Single | 27/6 | T:<br>29.6±12.8<br>C:<br>38.2±16.6 | TBI | GCS: | i：NI | No auditory sensory stimulation | SBP、DBP、RR、SPO2、PR、Temp | Auditory stimulation by family voices cannot increase or decrease the uniform trend in hemodynamics variables in comatose patients. | 8/10 |
| | | | | | | | | T：6.3 ± 2.2 | ii:Familiar voices:patient’s family members voice with his/her presentation, reassuring messages and a brief description of the incident that had occurred, | | | | |
| | | | | | | | | C:7.6 ± 3.5 | iii:2times/day | | | | |
| | | | | | | | | | iv:14days | | | | |
| | | | | | | | | | v:4min/time | | | | |

| | | | | | | | | | | | | | |
|---|---|---|---|---|---|---|---|---|---|---|---|---|---|
| 15 | Giesbrecht Puggina 2011 | Brazil | RCT | Single | NI | T: 44.7±18.7<br><br>C: 47.7±21.8 | CET;CRA ;Sedation; AS | GCS：3-8 | i：NI<br>ii：Music stimulation: A song vocalized by a therapist;The chosen music showed that a special moment in the history of that patient's life meant times of happiness.<br>iii:1time/day<br>iv:3days<br>v:NI | Play muted content for the same length as the experimental group's music | SBP、DBP、RR、SPO2、Temp | There were no significant differences in SBP、DBP、RR、SPO2、Temp between the music group and the control group after the inventation. | 7/10 |

T=trial group；C=control group；RCT=Randomized Controlled Trial

NI=No Information；MCS=minimally conscious state

TBI=Traumatic Brain Injury；HT=Head trauma

CET=cranial encephalic trauma；CRA = cardiac respiratory arrest

AS=abdominal surgery；GCS=Glasgow Coma Scale

SBP=Systolic Blood Pressure；DBP=Diastolic Blood Pressure

RR=Respiratory Rate；$SpO_2$=Peripheral Oxygen Saturation

PR=Pulse Rate；Temp=Temperature
