# Supplementary material for: Effects of auditory sensory stimulation on consciousness and physiological parameters among individuals with disorders of consciousness: a systematic review and meta-analysis
Source: Front Neurosci. 2026 Oct 28;20:1879145. doi: 10.3389/fnins.2026.1879145 (PMC13751540; doi:10.3389/fnins.2026.1879145)
Supplement: Supplementary file 5 [file table_3.pdf]

Supplementary Table 3. Univariate Meta-regression Analyses for GCS Effect Size

Moderator variable	Comparison	Number of studies	Coefficient (β)	Std. Err.	95% CI	P-value	tau²	Residual I²(%)	Adjusted R²(%)
risk of bias	high risk of bias vs. low risk of bias	11	-0.322	0.684	-1.868～1.224	0.648	0.944	85.14	-9.18
intervention type	linguistic auditory stimulation vs. non linguistic auditory stimulation	11	0.357	0.685	-1.192～1.906	0.615	0.990	83.06	-14.42
intervention frequency	≤1 time/day(reference)	3	reference	—	—	—	0.543	74.60	37.27
	2 times/day vs. ≤1 time/day	6	-0.401	0.607	-1.801～0.998	0.527	—	—	—
	3 times/day vs. ≤1 time/day	2	1.621	0.865	-0.374～3.616	0.098	—	—	—
intervention duration	per 1 day increase	11	0.025	0.053	-0.095～0.144	0.651	0.945	81.38	-9.20
intervention duration per session	0–15 minutes per session (reference)	7	reference	—	—	—	0.973	86.27	-12.53
	5–30 minutes per session vs. 0–15 minutes per session	3	-0.461	0.825	-2.364～1.442	0.592	—	—	—
	30–60 minutes per session vs. 0–15 minutes per session	1	1.279	1.472	-2.116～4.673	0.410	—	—	—
disease type	pure traumatic brain injury vs. mixed disease	11	0.731	0.677	-0.801～2.263	0.309	0.887	81.31	-2.51
types of control group interventions	conventional sound vs. no auditory stimuli	11	-0.783	0.648	-2.249～0.683	0.258	0.869	81.03	-0.46
